# The Path towards Endangered Species: Prehistoric Fisheries in Southeastern Brazil

**DOI:** 10.1371/journal.pone.0154476

**Published:** 2016-06-29

**Authors:** Mariana Samôr Lopes, Thayse Cristina Pereira Bertucci, Luciano Rapagnã, Rafael de Almeida Tubino, Cassiano Monteiro-Neto, Acácio Ribeiro Gomes Tomas, Maria Cristina Tenório, Tânia Lima, Rosa Souza, Jorge Domingo Carrillo-Briceño, Manuel Haimovici, Kita Macario, Carla Carvalho, Orangel Aguilera Socorro

**Affiliations:** 1 Universidade Federal Fluminense, Instituto de Biologia, Campus do Valonguinho, Outeiro São João Batista, s/n°.CEP: 24020–141, Niterói, Rio de Janeiro, Brasil; 2 Instituto de Pesca, Centro APTA Pescado Marinho, Av. Bartolomeu de Gusmão 192, Santos, São Paulo, CEP: 11030–906, Brasil; 3 Universidade Federal do Rio de Janeiro, Museu Nacional, Departamento de Antropologia. Quinta da Boa Vista, Rio de Janeiro, CEP: 20940–040, Brasil; 4 Palaeontological Institute and Museum, University of Zürich, Karl-Schmid-Strasse 4, Zürich 8006, Switzerland; 5 Universidade Federal do Rio Grande, Instituto de Oceanografia, Campus Carreiros, Av. Itália, Rio Grande, Rio Grande do Sul, CEP: 96201–900, Brasil; 6 Universidade Federal Fluminense, Instituto de Física, Campus da Praia Vermelha, Boa Viagem, CEP: 24210–310, Niterói, Rio de Janeiro, Brasil; New York State Museum, UNITED STATES

## Abstract

Brazilian shellmounds are archaeological sites with a high concentration of marine faunal remains. There are more than 2000 sites along the coast of Brazil that range in age from 8,720 to 985 cal BP. Here, we studied the ichthyoarchaeological remains (i.e., cranial/postcranial bones, otoliths, and teeth, among others) at 13 shellmounds on the southern coast of the state of Rio de Janeiro, which are located in coastal landscapes, including a sandy plain with coastal lagoons, rocky islands, islets and rocky bays. We identified patterns of similarity between shellmounds based on fish diversity, the ages of the assemblages, littoral geomorphology and prehistoric fisheries. Our new radiocarbon dating, based on otolith samples, was used for fishery characterization over time. A taxonomical study of the ichthyoarchaeological remains includes a diversity of 97 marine species, representing 37% of all modern species (i.e., 265 spp.) that have been documented along the coast of Rio de Janeiro state. This high fish diversity recovered from the shellmounds is clear evidence of well-developed prehistoric fishery activity that targeted sharks, rays and finfishes in a productive area influenced by coastal marine upwelling. The presence of adult and neonate shark, especially oceanic species, is here interpreted as evidence of prehistoric fisheries capacity for exploitation and possibly overexploitation in nursery areas. Various tools and strategies were used to capture finfish in seasonal fisheries, over rocky reef bottoms and in sandy littoral environments. Massive catches of whitemouth croaker, main target dermersal species of South Atlantic coast, show evidence of a reduction in body size of approximately 28% compared with modern fisheries. Fishery activity involving vulnerable species, especially in nursery areas, could mark the beginning of fish depletion along the southeastern Brazilian coast and the collapse of natural fish populations.

## Introduction

When investigating early archaeological settlements in Brazil, South America [1], the existence of an undisturbed marine fauna predating European colonization is expected. The archaeological evidence of prehistoric fisheries shows high abundance and diversity of marine faunal remains recovered from Brazilian shellmounds [2,3,4,5,6,7,8,9,10,11,12]. However, the main goal of these studies is to elucidate the archaeological context of fisher-gatherer settlements. The ichthyoarchaeological remains testify to well-developed fisheries for sharks, rays and finfishes, as well as to shellfish gathering and hunting of marine reptiles and mammals. Prehistoric subsistence fisheries could have been the cause of early differential disturbances in local fish fauna resources due to the assumed use of beach seines, gillnets, hook and line, traps and spearfishing.

A key study on prehistoric fisheries in the Caribbean [13] strongly supports the claim that overexploitation did not occur. However, the debate about fish depletion, overexploitation, extinction and environmental degradation in prehistoric and colonial times continues [14,15]. Prehistoric overfishing could be associated with local environmental degradation as a consequence of human uses of the landscape affecting the marine environment, community dynamics and spatially subsidized food webs [16,17,18,19], and overexploitation based exclusively on prehistoric fisheries might not have been the exclusive cause [15,20,21]. This hypothesis was widely discussed using evidence from comparative analyses of Pacific islands and channel ecosystems under archaeological investigation [22,23]. However, tropical Western Atlantic prehistoric fisheries differ in terms of Holocene paleoenvironments, fish assemblages and large-scale vulnerability at the time of prehistoric fisheries along the South American coast ([1]; S1 Appendix).

Prehistoric fishery exploitation patterns along the Atlantic coast of South America, from Brazil to Argentina are not well described. Brazilian shellmounds ranges from 8,720 to 985 years calibrated before present (cal BP) [1,24,25,26,27,28,29,30,31]. Previous ichthyological records from these archaeological sites were documented only by faunal lists without illustrative diagnostic species characters or museum catalog numbers to corroborate the species identifications. The present contribution is an attempt to improve our understanding of Rio de Janeiro’s shellmounds by providing new, detailed and accurate taxonomic lists and analyses of the relevant fish assemblages.

Contiguous with south Brazil and Uruguay, Argentinean ichthyoarchaeological sites, situated on the coast of San Matías Gulf in Patagonia (6,800 to 890 yr BP), show evidence of local prehistoric fisheries. The abundance of otoliths indicates that the predominant bony fish target was the whitemouth croaker, *Micropogonias furnieri* [32,33,34,35], a coastal finfish species also present in Brazilian coastal shellmounds.

Fishery tools, such as projectile points, manufactured mainly with bones are frequently recovered from the Brazilian shellmounds [30]. Moreover, evidence that allows us to infer the use of wood and vegetable fiber for the construction of fish traps, beach seines and or gillnets used for massive captures of large fish schools is not preserved. Indirect evidence of boat constructions based on lithic tools and records in detailed rock paintings reveal fluvial skillful navigators in Northeastern of Brazil [36]. In addition, the frequency of skeletal anomalies found in human remains recovered from the shellmounds, including auditory meatus exostoses, osteoarthritis, osteoarthrosis and other degenerative effects [37,38], is usually considered to be a marker of sailing and aquatic labor in cold waters [39,40,41], suggesting the engagement of those human populations in traditional fishery activity for subsistence. Stable isotope analyses of δ^14^C and δ^15^N in human skeletons from the shellmounds of Southern Brazil indicate a diet strongly dependent on marine resources [42]. Therefore, in agreement with previous research [43], the abundance and diversity of fish remains from the shellmounds provides unequivocal proof of fisheries that were able to operate in open waters over sandy and rocky bottoms.

Shellmounds are not necessarily horizontally stratified due to sequential periods of occupation, and the areas selected for specific activities may vary from the center to the periphery of the shellmound [44,45]. These sandy shellmounds are usually dome-shaped, and the archaeological variation in vertical section is based on changes in sediment texture and color, settlement size, abundance and diversity of mollusks, intercalation of sterile sandy layers (i.e., without ichthyoarchaeological remains) and evidence of cultural activities (e.g., burials and stoves). However, in contrast with archaeological evidence from settlements, in some shellmounds, the layers could not be distinguished [46].

The Rio de Janeiro shellmounds [1] are located in a coastal landscape characterized by sandy plains with coastal lagoons, rocky islands, islets and rocky bays [47]. These coastal areas are strongly influenced by seasonal upwellings, occurring during the austral summer [48,49,50,51], which increase marine productivity and the potential resources for fisheries.

Therefore, the main goal of this study is to highlight the fish diversity and faunal assemblage of ancient fisheries based on the skeletal remains deposited in the shellmounds along the southeastern Brazilian coast. Additionally, we correlate shark diversity and abundance in the shellmounds with the abilities of specific fisheries to exploit resources, which could lead to overexploitation. The groundfish catches were the result of multi-gear strategies in seasonal fisheries. Radiometric age, geochemical analyses and climate reconstruction of these ichthyoarchaeological remains [52,53,54,55] were used to supporting our findings.

### Historical accounts of ancient fisheries

Ichthyoarchaeological evidence and historical accounts from the Caribbean and tropical South America suggest that early prehistoric target species corresponded to the most accessible and vulnerable animals, such as sharks, large groupers [56,57,58], turtles [59,60] and sea mammals [61,62,63,64].

Colonial accounts from 1587 [56] on the semi-sedentary indigenous community that lived in Brazil during the early days of Portuguese colonization refer to the paleo-Indian fisheries’ expertise and their use of rich marine food supplies; the accounts additionally demonstrate a traditional knowledge of fish diversity, reproductive aggregation of fish, fishery areas and environmental relationships. Regarding fish diversity, a list of the 43 most important species of fish was reported accurately (Table 1) [56]. These fish records are in agreement with the prehistoric data presented here. Most of the historic narrative regarding fish captures concerns reproductive periods in coastal and estuarine areas, where bony fishes form compact aggregations along the littoral zone during the intertidal phase. The account ‘*curiosi rerum naturae*’ [56] refers to high marine fish abundance, especially during the summer on the coast of Salvador in the state of Bahia (northeast Brazil), when female bony fishes have large gonads. Additionally, the narrative talks about the ‘docile and very easily caught giant grouper on the beaches’, the large tarpon size, ‘longer than an Indian is tall’, and the ‘thousands of mullet caught during a single day fishing’, revealing a picture of the abundance of fishery resources [56,65]. All these historical narratives are in agreement with the fact that Brazilian neo-Indians were skilled fishermen, using arrows, marksmanship, and fishery lines with hooks; they built fish traps with wood and rocks in an intertidal beach and small nets for cooperative fisheries. These undeniable skills were possibly inherited from ancestral paleo-Indians who perfected the art of fishing [66].

**Table 1:** Brazilian colonial fish records [56].

Indigenous names	Probable species	Fishery tool and remarks
aragoagoay	*Pristis* sp.	hooks and spear
uperu, panapaná, socorí	shark	hooks and spear
beijupirá	*Rachycentron canadum*	hooks
tapyrsiçá	*Seriola lalandi*	hooks
camuropi	*Megalops atlanticus*	hooks, very large
piraquiroá	*Selene* sp.	hooks
carapitanga	*Lutjanus* sp.	hooks
canapú	*Epinephelus itajara*	hooks, tides stones and sticks tramp, very large, easy capture
cupá	*Cynoscion* sp.	hooks
guaripicú	*Scomberomorus* sp.	trolling lines
guiará	*Chaetodipterus faber*	hooks and beach seine
guris and urutús	Ariidae	hooks
caramurú	*Echidna* sp.	hands
jabubirá	Dasyatidae or Myliobatidae	hooks and beach seine
tacupapirema	*Micropogonias furnieri* or *Cynoscion acoupa*	hooks
bonitos	Carangidae	hooks
dourada	*Coryphaena hippurus*	hooks
caraoatá	*Thunnus* sp.	hooks
garoupas	*Epinephelus* sp.	hooks, very large
camurîs	*Centropomus* sp.	hooks
abróteas	*Urophycis brasiliensis*	hooks
ubaranas	*Elops saurus*	hooks
goaivicoára	*Conodon nobilis*	hooks
sororocas	*Scomberomorus maculatus*	hooks
timaçu	*Strongylura* sp.	used for bait
miracoaia	*Stellifer* sp. or *Bairdiella* sp.	hooks
maracuguara	*Balistes* sp.	hooks
paratîs	*Mugil curema*	sticks tramp and net during high tide
zabucai	*Selene* sp.	beach seine
tareîra	*Caranx hippos*	beach seine
coirimás	*Mugil liza*	beach seine
arabori	*Brevoortia aurea*	beach seine
carapebas	*Eucinostomus* sp.	beach seine
jaguaraçá	*Holocentrus adscensionis*	hooks, medicinal
piraçaque	*Conger* sp.	hooks, medicinal
bodiaens	*Scarus* sp.	hooks, medicinal
atucupá	*Cynoscion* sp.	hooks, medicinal
goayibicoati	Gobiidae	hooks, medicinal
uramaçâ	Paralichthyidae	hooks, medicinal
baiacú	*Lagocephalus* sp.	fishes that producing poisoning
piraquiroâ	*Chilomycterus antillarum*	fishes that producing poisoning
aimoré	Gobiidae	fishes that producing poisoning

The first ichthyofaunal list (indigenous name) documented from the Brazilian coast.

Shellmounds are the best testimonial resource for understanding the paleo-Indian fishery activities. However, most of the original context of Brazilian shellmounds was destroyed due to the use of mollusk shells to produce lime and fertilizers for paving of roads and streets, construction of forts, colonial houses, churches, among others applications. This occurred starting in 1549 in the area of Salvador, Bahia and other colonial settlements along the Brazilian coast and continued until the 1960s, when archaeological shellmounds became protected under Brazilian federal law.

### Geographic setting

From south to north, the landscape of the Rio de Janeiro coast is characterized by the presence of a crystalline shield (Serra do Mar relief), with plenty of high (approximately 1,200 m) mountain scarps parallel to the Atlantic Ocean in the vicinity of Angra dos Reis [67]. The area is characterized by short rivers flowing to the coast and fluviomarine plains in an embayment (i.e., Ribeira Bay), a jagged coastline, with small peninsulas and several rocky islets [68]. The beaches and the sandy stretches are not developed, and the shellmounds are predominantly located over the rocky islets in an area dominated by mangrove flood plains. Small submarine channels approximately 6 to 10 m deep characterize the coastal bathymetry, and near Ilha Grande bay, the depth reaches approximately 30 m. Ilha Grande is a massive structural island with fairly rugged relief; it is isolated from the mainland by a channel approximately two kilometers wide.

The Itaipú-Camboinhas region is located on the oceanfront of Niterói and has sandy beaches dominated by dunes and sandy bars, which separate the sea from Piratininga and Itaipu coastal lagoons. The semicircular sandy beach has depths of approximately 3 to 16 m that extend almost 1,000 m offshore. The landscape has a mountainous relief aligned in the SW-NE direction [47]. The Camboinhas shellmound is located over a sand dune, near the coastal line and the tidal channel of Itaipu Lagoon.

The Saquarema region is located in a landscape characterized by a crystalline rock relief, which separates the two major drainage basins that feed the Saquarema lagoon complex [47,68]. The sandy shore is shallow but exposed to high-energy coastal wind and waves. The area has the highest concentration of shellmounds in Rio de Janeiro; they are distributed along the sandy coastal plain of the inland sandbanks, facing the lagoon (e.g., Beirada, Manitiba, Ponte do Girau and Saquarema shellmounds).

In Arraial do Cabo, the structural NE-SW trend is characterized by a metamorphic basement (i.e., Pontal de Atalaia), rising up to 172 m high, and the adjacent Cabo Frio Island, an igneous alkaline rock (syenite, trachyte and breccia) with altitudes of approximately 380 m [69]. The Usiminas shellmound is located in the Cabo Frio Island, 50 m above sea level, facing the coastal plain, where the water depth is approximately 5 m. In contrast, the oceanfront cliffs on the opposite coast reach 50 m in depth near the coastline. Additionally, the Ilha do Cabo Frio shellmound is located on a small sandy beach characterized by an active dune that faces towards the landscape, associated with the outcrop layers that overlap the Cabo Frio beach rock [65,70]. Part of the lower layer of this shellmound is located below sea level [29].

## Materials and Methods

Selected shellmounds from the southeastern coast of Rio de Janeiro State, Brazil have three main features: **(1)**, the potential marine influence of the Cabo Frio upwelling system (i.e., seasonal oceanographic mixing of South Atlantic Central Water, Subtropical Shell Water and the Brazilian Current, increasing biological productivity); **(2)**, the marine environment (i.e., shallow waters, coastal lagoons and a rocky bottom); and **(3)**, coastal geomorphology (i.e., sandy coastal plains, rocky islands and rocky bays).

**Fig 1:**
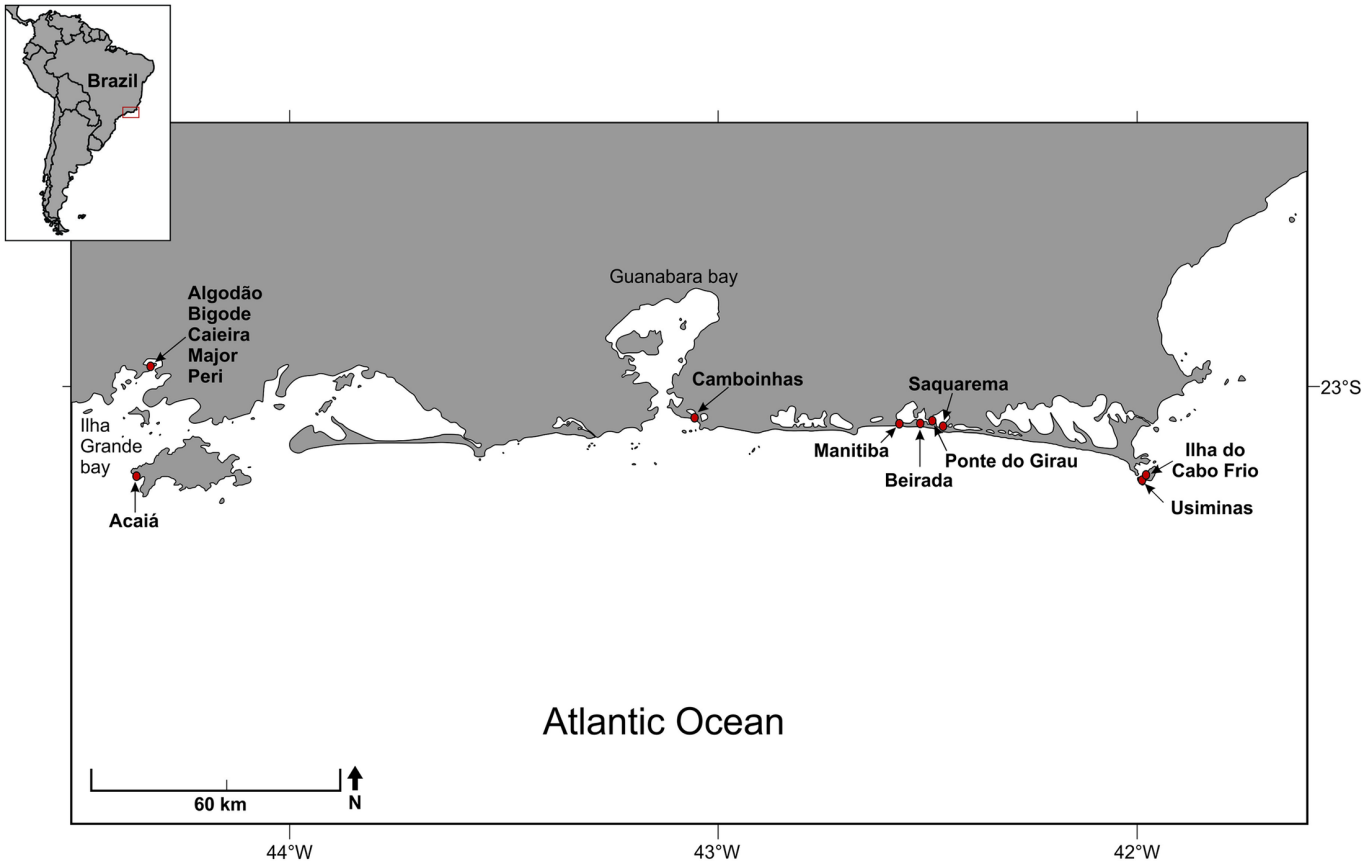
Study area along the southeastern Brazilian coast showing shellmound locations.

The shellmounds included in this study are the following: the Usiminas shellmound [71], on a rocky settlement, and the Ilha do Cabo Frio shellmound [72], on a sandy beach on Cabo Frio Island (23°00' 18" S, 42°00' 20" W); Saquarema [72], Beirada [46], Manitiba [73] and Ponte do Girau [74] shellmounds, on a sandy coastal plain with coastal lagoons in the Saquarema lagoon complex (22°55' 66" S, 42°29' 00" W); Camboinhas shellmound [75], on a sandy coastal plain with coastal lagoons in the oceanic region of Niterói (22°57' 54" S, 44°02' 53" W); Algodão, Major, Bigode, Caieira and Peri shellmounds [76], on rocky islets and coastal rocky bays in the Ribeira Bay, Angra dos Reis (22°55' 48" S, 44°20' 48" W); and Acaiá shellmound (personal communication of an unpublished manuscript: Tenório, M.C. ‘Os sambaquieiros e a gruta do Acaiá: Reconstituição do processo de formação de um sítio’), on a rocky island in the oceanfront of Ilha Grande (Fig 1).

We studied all the specimens deposited in the ichthyoarchaeological collection of Museu Nacional, Universidade Federal do Rio de Janeiro (MN-UFRJ), Rio de Janeiro, Brazil, and their use for this research was authorized by the collection managers, who are coauthors of this study (MCT and TL).

We have organized a referential species collection based on 679 diagnostic osteological and dental characters (details in S2 Appendix). Quantitative analyses of fish specimens and species recovered from each shellmound are necessary for accurate data interpretation. However, different archaeological methods were used in these shellmounds during excavation conducted by UFRJ archaeologists between 1981 and 2005. The methods included sieving techniques, removing material and curatorial processing. These and others questions regarding the repository, catalog and samples labels will require more detailed assessment, which is beyond of the scope of this study. Consequently, the fish diversity analyzed here is based on a qualitative study and the frequencies of species by shellmound.

All relevant specific characteristics of examined species were identified based on comparative anatomy, using 39 shark and ray specimens (among teeth, vertebrae and spines) housed at the Universidade Estadual do Rio de Janeiro (UERJ) and using at least 115 otoliths and 21 dry finfish individual skeletons housed at the Universidade Federal Fluminense (UFF) (details in S3 Appendix). All structures are in a good state of conservation and relevant specific osteological characteristics were also identified based on extensive bibliographic review.

Photographs of the otoliths were taken with a Leica M205A multifocal stereomicroscope. Photographs of bones, sharks and rays teeth were taken using a digital microscope and digital camera. A complete atlas of the most common diagnostic teeth, otoliths and bones recovered and observed from shellmound collections is included in the plates of the present paper.

Cluster analysis was performed under the Paleontological Statistics Software (PAST, version 2.17c) on Q-mode (i.e., grouping variables) to analyze shellmound similarity patterns. This exploratory technique identifies the relationships and patterns among multiple variables across samples and has been applied in a wide range of scientific fields, such as marine and fisheries ecology [77,78,79,80]. The analysis was based on the presence and absence of fish assemblages in the shellmounds, archaeological site ages, littoral geomorphology and prehistoric fisheries. The unweighted pair-group average (UPGMA) algorithm was used with the Bray-Curtis similarity-association matrix of [81].

Estimates of shark-body total length (TL) were based on 660 isolated vertebrae, using a unique linear regression equation for each species: *Carcharias taurus* TL = 36.786 +10.753 CR [82], *Sphyrna lewini* TL = 4.51 + 23.64 CR [83], *Carcharhinus brevipinna* CD = 0.0159 PCL– 0.1285, PCL = 0.799 TL– 9.07 [84] and *Carcharodon carcharias* FL = 21+ 11.8 CR, FL = 0.94 TL– 5.74 [85], where TL is the total length, FL is the fork length, PCL the precaudal length, CD the vertebral centrum diameter and CR the vertebral centrum radius.

The main teleostean fish target, based on the frequency observed in the shellmounds, was the whitemouth croaker, *Micropogonias furnieri* [86], which was present in all shellmounds with the exception of the Usiminas and Ilha do Cabo Frio shellmounds, both on Cabo Frio Island. The life history of *M*. *furnieri* in the Western Atlantic Ocean is well known [87,88], and they can be found in commercial [89,90] and local artisanal fisheries [77,91]. We used this species to interpret and compare changes between past and present coastal fisheries on the southeastern Brazilian coast. We tested for differences between the medians of length frequency distributions (Student’s t-test, PAST software v. 3.7) after checking for normality and homogeneity of variances.

A total of 5,532 archaeological whitemouth croaker, *Micropogonias furnieri*, otoliths were measured using digital callipers. These specimens were distributed among the shellmounds as follows: Ponte do Girau (376 specimens), Beirada (2,541 specimens), Manitiba (1,372 specimens), Algodão (1,148 specimens) and Camboinhas (95 specimens). Fish total lengths (TL) were calculated based on the archaeological otolith total length (OL) using the following equation, which we derived from a regression analysis: TL = 24.34 + 22.57 OL (r = 0.988, n = 93). To compare the estimates of body size length data between shellmound samples, we performed a nonparametric variance analysis of medians (Kruskal-Wallis test) and an *a posteriori* test of the shellmound context, analyzing the localities and related environmental factors using the free software R [92].

We chose not to use the South American Western Atlantic fish records [88]; instead, specific references of modern fish diversity and abundance in the littoral areas close to the shellmounds of Rio de Janeiro were obtained from Arraial do Cabo [58,93,94], Itaipu [77,95] and Ribeira Bay in Angra dos Reis [96], and these were used in comparisons with fishery records.

Data from the Brazilian fishery survey of whitemouth croaker in the Itaipu modern fisheries (i.e., artisanal and semi-industrial) are based on records compiled between 2000 and 2004 [77] from the Angra dos Reis Bay from semi-industrial fisheries captures reported between 2013 and 2014. At Guanabara Bay, a fishery study was conducted using a bottom trawl between 2005 and 2007 [91].

The samples for radiochronology were prepared and analyzed at the Radiocarbon Laboratory of the Universidade Federal Fluminense (LAC-UFF). The otolith samples were chemically treated with HCl and converted to CO_2_ by hydrolysis with H_3_PO_4_. Graphitized samples were placed in the 40-sample wheel of the SNICS ion source and measured in an NEC 250 kV Single Stage Accelerator System (SSAMS) [97]. Typical currents were 50 μA^12^C^-1^ (measured at the low energy Faraday cup). Graphite standard and calcite blanks yielded average ^14^C/^13^C ratios of 6 x10^-13^ and 7x10^-13^, respectively. The average machine background was approximately 50 kHz for the unprocessed graphite, while the average precision ranged from 0.3 to 0.5%. Data analyses were carried out on LACAMS software developed at the Physics Institute of Universidade Federal Fluminense [98]. Calibration of otolith radiocarbon dates was performed with OxCal software v 4.2.3 from the Oxford University [99], using the Marine13 curve [100] in the 2-sigma range with an offset for local marine reservoir corrections (ΔR ^14^C years) according to the following shellmound localities: Saquarema [53], Manitiba [55] and Southeastern Brazil [101]. Cross-comparisons were conducted in the Beta Analytic Inc. laboratory, following standard protocols.

We exclusively used the fish otolith radiocarbon dates to determine ages for chronological interpretation of the fish assemblages of most shellmounds, except for those from the Cabo Frio Island, where otoliths of whitemouth croaker could not be recovered; additionally, a few otoliths from Ilha Grande could not be dated. Consequently, we refer only to ages that were not derived from otolith samples for the Usiminas, Ilha do Cabo Frio and Acaiá shellmounds. The age ranges of those sites are based on charcoal and shell data ([29,102]; personal communication).

## Results

**Fig 2:**
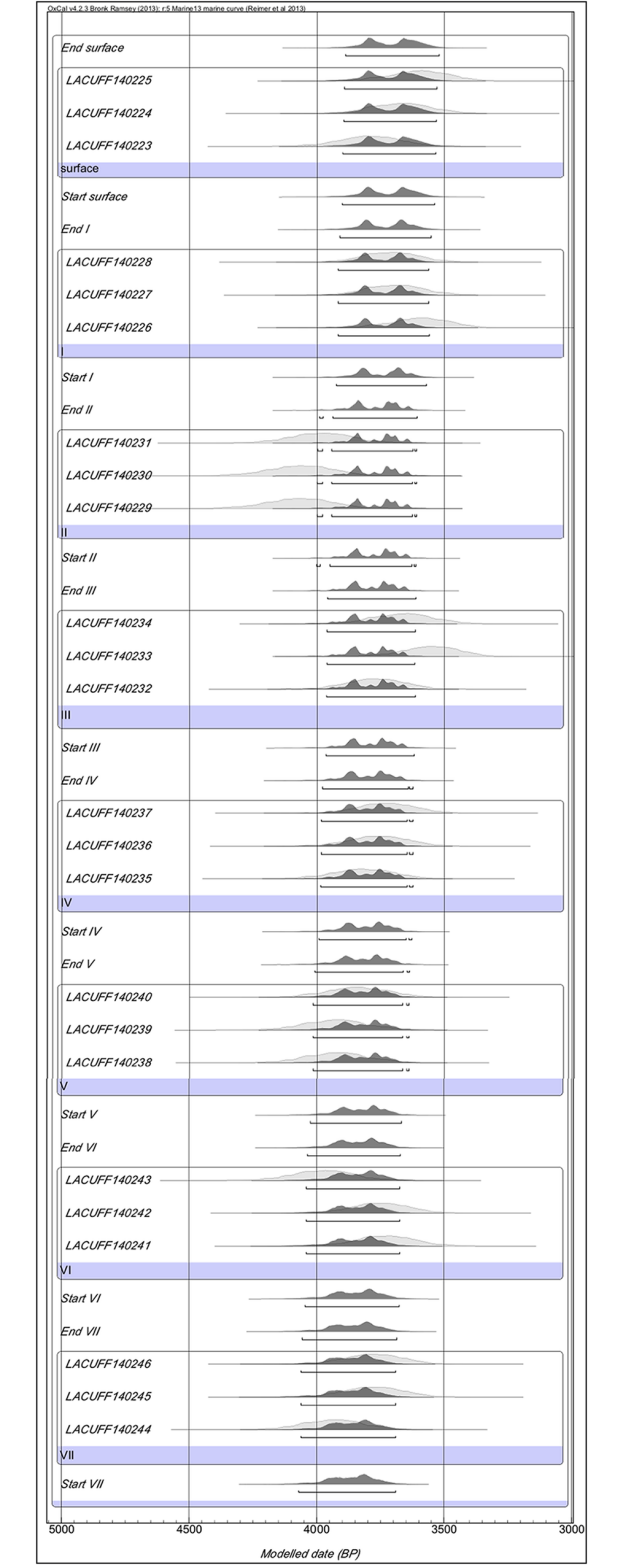
Modeled age based on whitemouth croaker otolith samples from multiple archaeological layers of Manitiba in Saquarema using OxCal v 4.2.3 [99]. This figure shows ages obtained by radiochronology (vertical lines). The error bar, represented by the horizontal line, shows a range of ages that spans approximately 370 years across the seven archaeostratigraphic sections, hindering a precise dating of the layers.

Shellmound radiocarbon dating based on otoliths and the identified fish assemblages are shown in Table 2. Manitiba serves as an example of a multilayer shellmound (i.e., seven archaeostratigraphic layers in 2.2 m of depth) and demonstrates the difficulty in distinguishing radiocarbon age differences between successive layers; here, the calibration curve associated with the error bar and the reservoir effect cause the age range to overlap (Fig 2). This inhibits inferring a chronological sequence for fisheries in shallow shellmounds. We, therefore, use individual shellmound dating as a marker of settlement period.

**Table 2:** Ichthyoarchaeological records from the Rio de Janeiro shellmounds (Saquarema, Cabo Frio, Niterói, Ilha Grande and Angra dos Reis).

Region		SAQUAREMA				NITEROI	ANGRA DOS REIS					ILHA GRANDE	ILHA DO CABO FRIO			
Shellmounds		Beirada	Saquarema	Manitiba	Girau	Camboinhas	Algodão	Bigode	Caieira	Peri	Major	Acaiá	Usiminas	Ilha do Cabo Frio	Structures	Figures
Geomorphology		Sandy coastal plain and coastal lagoons				Sandy beach	Rocky bottom and rocky islet					Rocky island		Sandy beach		
Radiocarbon age ranges		3035 to 5595 cal BP	2100 to 4200 cal BP	3695 to 4515 cal BP	3730 to 4525 cal BP	4160 to 4960 cal BP	2345 to 4414 cal BP	3223 to 3525 cal BP	1875 to 2175 cal BP	890 to 1140 cal BP	675 to 900 cal BP	2760 to 2930 cal BP*	1265 to 1765 cal BP*	2710 to 3290 cal BP*		
**Chondrichthyes**																
Odontaspididae	*Carcharias taurus*			**·**		**·**	**·**	**·**	**·**	**·**	**·**	**·**	**·**	**·**	teeth, vertebrae	Fig. 8.1; 10.1; 10.4
Alopidae	*Alopias superciliosus*		**·**												teeth	Fig. 8.2
Lamnidae	*Carcharodon carcharias*	**·**	**·**			**·**	**·**				**·**	**·**	**·**	**·**	teeth, vertebrae	Fig. 7.1; 8.3; 10.2; 10.3
	*Isurus oxyrinchus*						**·**					**·**	**·**	**·**	teeth	Fig. 8.4
	*Lamna nasus*						**·**	**·**	**·**	**·**	**·**		**·**	**·**	teeth	Fig. 8.5
Carcharhinidae	*Carcharhinus acronotus*						**·**		**·**		**·**			**·**	teeth	Fig. 8.6
	*Carcharhinus altimus*											**·**			teeth	Fig. 7.2
	*Carcharhinus brevipinna*	**·**	**·**			**·**	**·**					**·**	**·**	**·**	vertebrae	Fig. 10.5-10.7
	*Carcharhinus leucas*													**·**	teeth	Fig. 7.3
	*Carcharhinus limbatus*						**·**	**·**	**·**	**·**	**·**	**·**			teeth	Fig.8.8
	*Carcharhinus obscurus*	**·**	**·**									**·**			teeth.	
	*Carcharhinus plumbeus*	**·**					**·**		**·**	**·**	**·**		**·**		teeth	Fig.7.4; 8.9
	*Carcharhinus porosus*						**·**	**·**	**·**	**·**	**·**				teeth	
	*Carcharhinus priscus*										**·**		**·**	**·**	teeth	
	*Carcharhinus* sp.	**·**	**·**			**·**	**·**					**·**	**·**	**·**	teeth	Fig. 10.8; 10.10-10.12
	*Galeocerdo cuvier*						**·**		**·**	**·**	**·**	**·**	**·**	**·**	teeth	Fig. 7.5; 8.10
	*Negaprion brevirostris*		**·**				**·**	**·**	**·**	**·**	**·**	**·**		**·**	teeth	Fig. 8.11
	*Rhizoprionodon lalandii*						**·**	**·**		**·**	**·**				teeth	Fig. 8.12
	*Rhizoprionodon porosus*						**·**	**·**			**·**				teeth	Fig. 8.14
	*Rhizoprionodon* sp.												**·**		vertebrae	Fig. 10.9
Sphyrnidae	*Sphyrna mokarran*		**·**				**·**		**·**		**·**				teeth	Fig. 7.6; 8.7; 8.15
	*Sphyrna zygaena*						**·**				**·**		**·**		teeth	Fig. 8.16
	*Sphyrna* sp.					**·**			**·**	**·**		**·**	**·**		teeth, vert.	Fig. 10.13; 10.14
Pristidae	*Pristis* sp.		**·**				**·**					**·**	**·**		vertebrae	Fig. 9.1; 9.2
Dasyatidae	*Dasyatis centroura*	**·**	**·**			**·**									caudal spine	Fig. 9.6
Myliobatidae	*Aetobatus narinari*					**·**	**·**					**·**	**·**	**·**	tooth	Fig. 9.7
Rhinopteridae	*Rhinoptera* sp.						**·**					**·**		**·**	tooth	Fig. 9.3; 9.4
**Osteichthyes**																
Albulidae	*Albula nemoptera*						**·**								otolith	Fig. 11.1
Ariidae	*Aspistor* sp.			**·**											bones	Fig. 13.1
	*Bagre bagre*						**·**				**·**				otolith	
	*Bagre marinus*						**·**	**·**		**·**		**·**			otolith, bones	Fig. 11.2; 13.2
	*Bagres* sp.			**·**											bones	
	*Cathorops* sp.			**·**											bones	Fig. 13.3
	*Genidens genidens*			**·**		**·**						**·**			otolith, bone	Fig. 11.3
	*Genidens barbus*			**·**											bones	Fig. 13.4
	*Genidens* sp.			**·**											bones	Fig. 13.5-13.8
	gen. sp. ind.				**·**								**·**		otolith	
Hemiramphidae	*Hemiramphus* sp.								**·**						otolith	Fig. 11.4; 11.5
Holocentridae	*Sargocentrum* sp.											**·**			bones	Fig. 13.9
Centropomidae	*Centropomus* sp.											**·**	**·**		bones	
	*Centropomus ensiferus*						**·**				**·**			**·**	otolith	Fig. 11.6
	*Centropomus undecimalis*						**·**						**·**		otolith, bones	Fig. 11.7; 13.11
	*Centropomus parallelus*			**·**									**·**		bones	Fig. 13.10
Serranidae	*Epinephelus morio*						**·**								otolith	
	*Epinephelus marginatus*												**·**		otolith	Fig. 11.9
	*Epinephelus* sp.						**·**				**·**	**·**	**·**	**·**	otolith, bones	Fig. 11.8; 13.12; 13.13
	*Mycteroperca* sp.												**·**		bones	Fig. 13.14
Coryphaenidae	*Coryphaena hippurus*											**·**			bones	
Carangidae	*Caranx hippos*					**·**						**·**	**·**		bones	
	*Caranx* sp.			**·**		**·**						**·**			bones	Fig. 13.15; 13.16
	*Oligoplites saurus*											**·**			bones	Fig. 14.1
	*Selar crumenophthalmus*											**·**			bones	
	*Selene vomer*											**·**	**·**		bones	Fig. 13.17; 13.18
Lutjanidae	*Lutjanus synagris*						**·**	**·**	**·**	**·**	**·**				otolith	Fig. 11.10
	*Lutjanus* sp.						**·**	**·**	**·**		**·**			**·**	otolith	Fig. 11.11
	*Ocyurus chrysurus*												**·**		otolith	Fig.11.12
	gen., sp. ind.													**·**	bones	
Gerreidae	*Diapterus rhombeus*						**·**		**·**	**·**	**·**				otolith	Fig. 11.13
	*Gerres cinereus*						**·**								otolith	Fig. 11.16
	gen., sp. ind.						**·**		**·**		**·**				bones	Fig. 14.3
Haemulidae	*Anisostremus virginicus*						**·**	**·**	**·**	**·**	**·**		**·**		otolith	Fig. 11.17
	*Anisostremus* sp.						**·**								bones	Fig. 14.4; 14.5
	*Haemulon aurolineatum*						**·**						**·**		otolith	Fig. 11.18
	*Haemulon sciurus*						**·**								otolith	Fig. 11.19
	*Haemulon steindachneri*						**·**		**·**				**·**		otolith	Fig. 11.20
	*Haemulon* sp.									**·**	**·**				otolith	
	*Orthopristis ruber*								**·**		**·**				otolith	
Sparidae	*Archosargus rhomboidalis*						**·**				**·**				otolith	Fig. 11.21
	*Archosargus* sp.						**·**					**·**		**·**	otolith, bones	Fig. 11.22; 14.7
	*Diplodus* sp.						**·**				**·**	**·**			otolith, bones	Fig. 11.14; 11.15; 14.6
	*Pagrus pagrus*												**·**		bones	
Sciaenidae	*Bairdiella ronchus*						**·**	**·**	**·**						otolith	Fig. 11.23
	*Cynoscion acoupa*			**·**											otolith	Fig. 11.26
	*Cynoscion jamaicensis*											**·**			otolith	Fig. 11.24
	*Cynoscion microlepidotus*				**·**		**·**	**·**	**·**	**·**	**·**				otolith	Fig. 11.25
	*Larimus breviceps*						**·**		**·**						otolith	Fig. 11.27
	*Micropogonias furnieri*	**·**	**·**	**·**	**·**	**·**	**·**	**·**	**·**	**·**	**·**	**·**			otolith, bones	Fig. 11.28
	*Pareques acuminatus*			**·**											otolith	Fig. 11.29
	*Pogonias cromis*	**·**	**·**	**·**	**·**		**·**							**·**	otolith, bones	Fig. 12.1; 14.9
	*Umbrina coroides*													**·**	otolith	Fig. 12.2
Mugilidae	*Mugil liza*						**·**	**·**	**·**	**·**	**·**				otolith	Fig. 12.3
	*Mugil* sp.			**·**											bones	Fig. 14.10
Labridae	*Bodianus rufus*											**·**		**·**	bones	Fig. 14.11; 14.12
Scaridae	*Scarus* sp.						**·**					**·**		**·**	bones	Fig. 14.14; 15.1; 15.2; 15.5
	*Sparisoma* sp.											**·**	**·**	**·**	bones	Fig. 14.8; 14.13; 15.3;15.4; 15.13
Trichiuridae	*Trichiurus lepturus*											**·**			bones	Fig. 15.6
Scombridae	*Katsuwonus pelamis*											**·**	**·**	**·**	bones	Fig. 15.7; 15.8
	*Scomberomorus* sp.											**·**	**·**		bones	Fig. 15.9
Istiophoridae	*Istiophorus albicans*											**·**	**·**		bones	Fig. 15.10
Ephippidae	*Chaetodipterus faber*		**·**										**·**		bones	
Sphyraenidae	*Sphyraena barracuda*												**·**		bones	Fig. 15.11
	*Sphyraena guachancho*						**·**				**·**				otolith	Fig. 12.4
	*Sphyraena* sp.								**·**		**·**	**·**	**·**	**·**	otolith, bones	
Hyporhamphidae	*Hyporhamphus unifasciatus*						**·**		**·**						otolith	Fig. 12.5
Tetraodontidae	*Lagocephalus laevigatus*						**·**					**·**		**·**	bones	Fig. 15.12
Diodontidae	*Chilomycterus spinosus*						**·**								bones	Fig. 15.15
	*Diodon* sp.												**·**		bones	Fig. 15.14

Ages are based on radiocarbon analyses of otoliths (*M*. *furnieri*). Ages with asterisks indicate that otolith radiocarbons ages were not available and charcoal- and shell-derived ages were used instead ([29,102]; personal communication).

A total of 97 fish species have been identified from the Rio de Janeiro shellmounds, representing 37% of the total (265 spp.) modern species recorded from the Rio de Janeiro coast [e.g., Arraial do Cabo (135 spp.), Itaipu (165 spp.) and Angra dos Reis (139 spp.)]. Table 2 shows the occurrence in archaeological sites of the great white shark, *Carcharodon carcharias*, the sand tiger shark, *Carcharias taurus*, the porbeagle shark, *Lamna nasus*, the lemon shark, *Negaprion brevirostris*, the tiger shark, *Galeocerdo cuvier*, and a large diversity of other sharks (such as Carcharhinidae, Sphyrnidae, and other coastal species) in the rocky islet shellmounds of Angra dos Reis (Ribeira Bay: Algodão, Bigode, Caieira, Major and Peri sites), Ilha Grande (Acaiá) and Cabo Frio Island (Usiminas and Ilha do Cabo Frio). The data also include the occurrence of other rarer shark species found in the shellmounds located over sandy coasts and coastal lagoons. These remains (i.e., shark teeth and vertebrae) have been recovered in 100% of the analyzed shellmound samples, especially from those associated with rocky islands and islets. The remains of the spotted eagle ray, *Aetobatus narinari*, were the most frequent from the ray group (batoids). The frequency of bony fish remains suggest that some species of groundfish, inhabitants of sandy and muddy bottom or rocky spots, such as croakers (Sciaenidae), snappers (Lutjanidae), grunts (Haemulidae), groupers (Serranidae) and snooks (Centropomidae), were common target species. Sea catfish (Ariidae) and mullet (Mugilidae) species were frequent in localities with sandy and muddy bottoms associated with coastal lagoons. Rocky reef fishes, which are mostly durophagous (i.e., feeding on mollusks, echinoids and crabs), are represented by species of parrotfish (Scaridae), pufferfish (Tetraodontidae) and porcupinefish (Diodontidae), and their remains were particularly frequent in localities associated with rocky islands. Pelagic fish, including bluefish (Pomatomidae), sailfish (Istiophoridae), dolphinfish (Coryphaenidae), jacks (Carangidae), mackerel and tuna (Scombridae), were frequent in the shellmounds located in coastal and rocky islands oceanfront areas.

**Fig 3:**
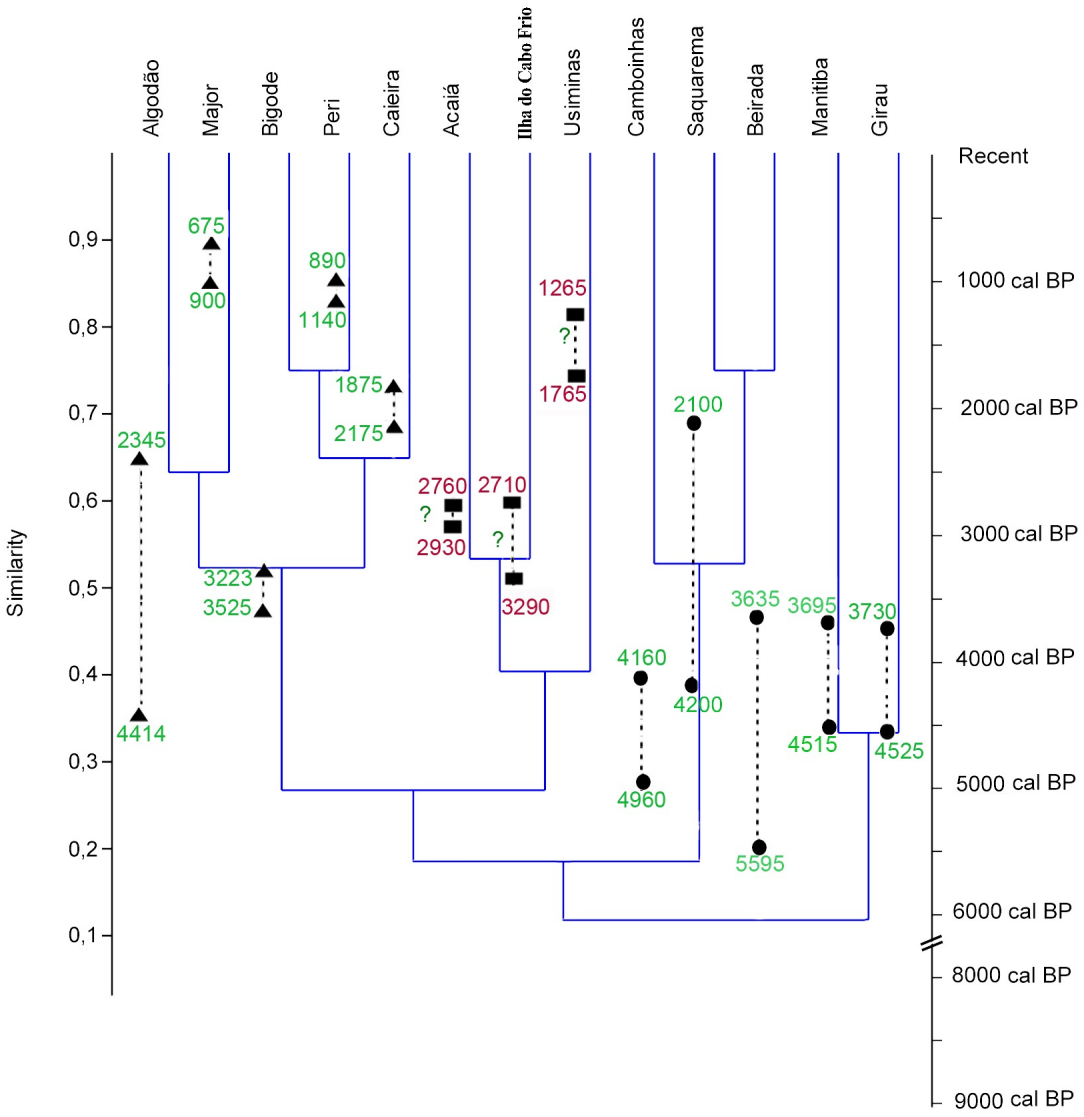
Cluster analysis of shellmound similarities. Age ranges (green letters) based on otolith data. Calibration was performed using Marine13 [100] in the 2-sigma range. The overall ΔR was found to be -56.5 to 120.5 [101]. The results at specific sites are as follows: ΔR Saquarema: -272 to -8 [53] and ΔR Manitiba: -224 to 60 [55]. Question marks indicate that radiocarbon dating of otoliths was not available; consequently, we plot shellmound age ranges (red numbers) based on charcoal and shell analyses ([29,102]; personal communication).

Based on the analyzed fish assemblages from shellmounds in Rio de Janeiro State, the nearshore fishery remains analyzed here were deposited 5,595 cal BP in the lagoon region of Saquarema and in the oceanfront region of Niterói (Fig 3, black circles). They were characterized by catches of coastal species associated with sandy bottoms and coastal lagoons. The fish could be accessible using beach seines during reproductive aggregation and spawning. These schools of fish include croaker, drum, catfish, mullet and snook. Later, approximately 4,414 cal BP, fishery activity records suggest a targeting of pelagic resources in protected rocky bays and around coastal rocky islets (Fig 3, black triangle). Rocky reef fishes were also a common target, and advances in artisanal fishery and multi-gear techniques remained successful until colonial times. At least since 3,290 cal BP, the fish assemblage recovery from the shellmounds located on the oceanic islands of Cabo Frio and Ilha Grande (Fig 3, black square) suggests a clear predominance of pelagic fisheries and a secondary use of rocky reef species.

The estimation of shark body size (TL) (Table 3) indicates that the spinner shark, *Carcharhinus brevipinna*, ranged from 44.3 to 263.1 cm; the sand tiger shark, *Carcharias taurus*, ranged from 79.3 to 192.2 cm; unidentified shark species of the genus *Carcharhinus* ranged from 38.8 to 115.1 cm; the hammerhead shark, *Sphyrna* sp., ranged from 10.1 to 40.0 cm; and the great white shark, *Carcharodon carcharias*, ranged from 88.2 to 249.6 cm.

**Table 3:** Summary of shark species and body size data recorded from the Rio de Janeiro shellmounds.

Common name	Species	N	Shellmound localities	Body size range (cm)	Mean Size (cm)
Spinner shark	*Carcharhinus brevipinna*	500	ALG, CAM, USI	44.3–263.1	124.1 ± 55.1
Shark	*Carcharhinus* sp.	87	USI	38.8–115.1	64.6 ± 15.1
Great white shark	*Carcharodon carcharias*	12	ALG, CAM, USI	88.2–249.6	153.3 ± 58.2
Sand tiger shark	*Carcharias taurus*	12	ALG, CAM, USI	79.3–192.2	122.1 ± 31.2
Hammerthead	*Shyrpna* sp.	49	ALG, CAM, USI	10.1–40.0	20.2 ± 5.1

(N) Number of ichthyoarchaeological remains of vertebrae. Shellmound localities: (ALG) Algodão, (CAM) Camboinhas, (USI) Usiminas.

**Fig 4:**
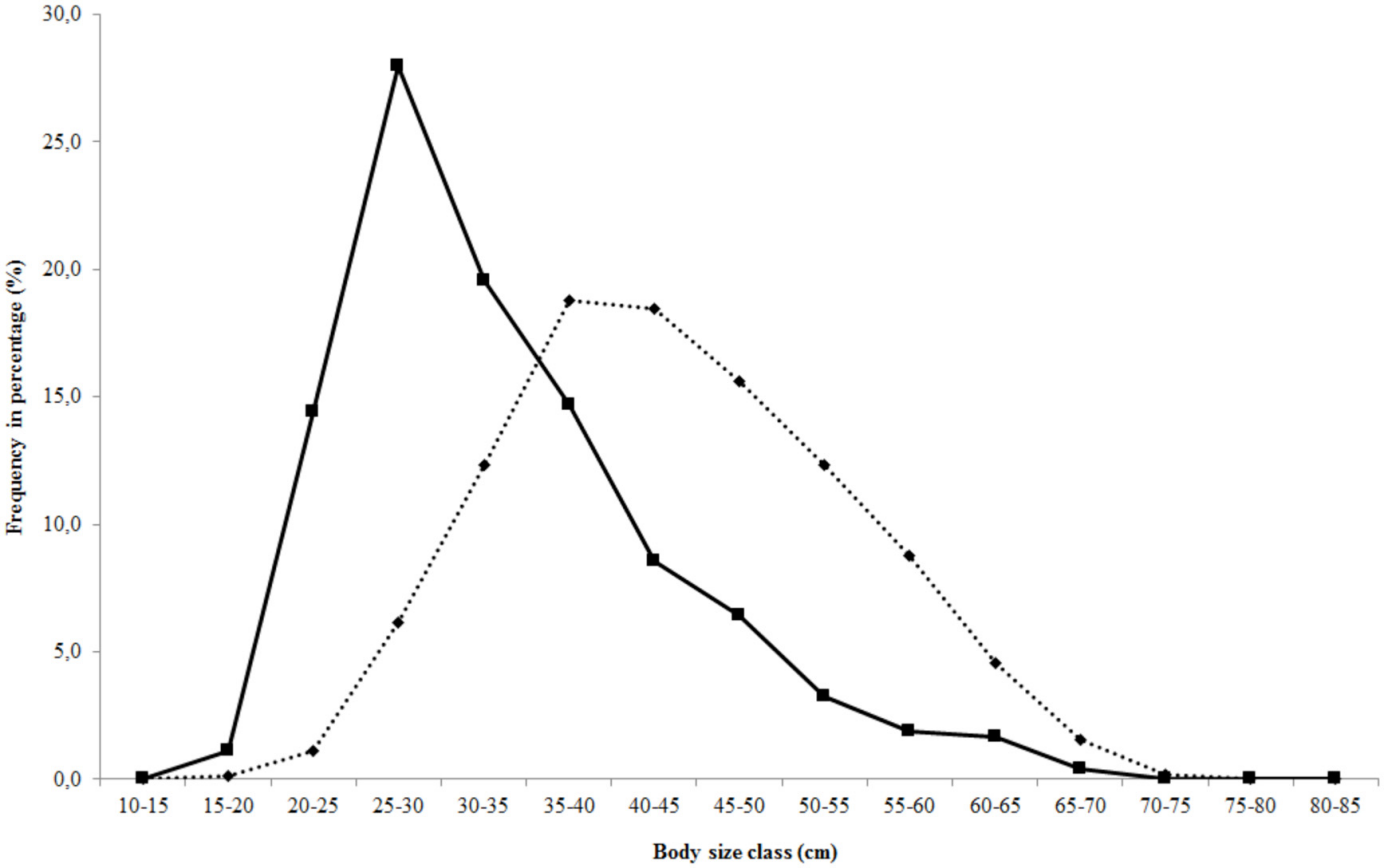
Frequency of body size classes of whitemouth croaker, *M*. *furnieri*. Ichthyoarchaeological (dashed line, N = 5,532) and modern fisheries (solid line, N = 3,914).

Estimation of the body size distribution of whitemouth croaker, *Micropogonias furnieri* [range: 17.8 to 84.8 cm TL in all shellmounds (mean length ± SD: 43.3 ± 9.9 cm)]. These ichthyoarchaeological data overlap the modern size distributions from modern fisheries in Itaipu and Angra dos Reis in Rio de Janeiro State (Fig 4). However, the body size distribution of whitemouth croaker catches from prehistoric fisheries shows a probabilistic tendency toward higher frequencies of large specimens, resulting in an estimated 28% reduction in body size based on modern catches.

**Fig 5:**
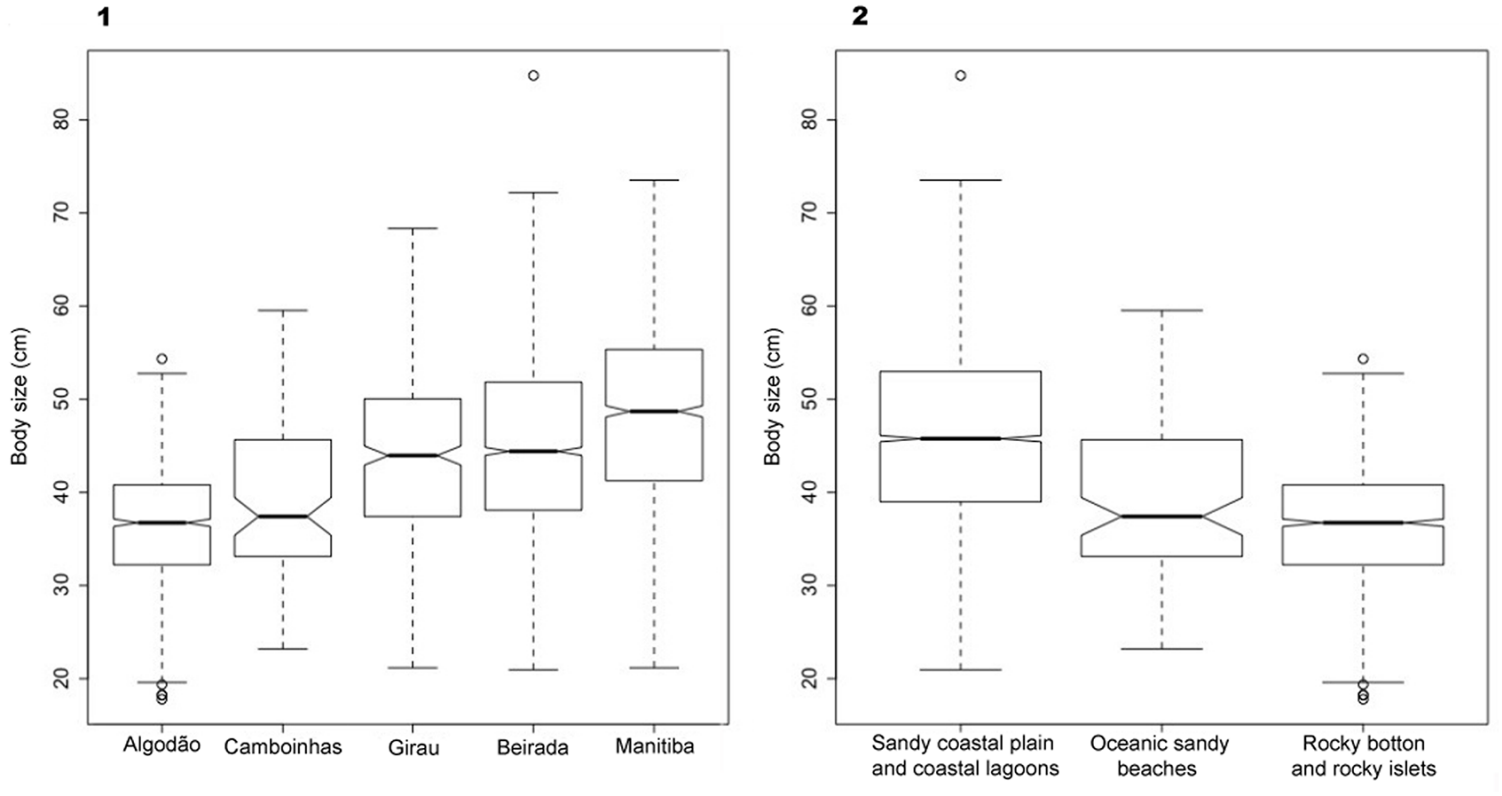
ANOVA analyses (Kruskal-Wallis) of medians based on estimates of body size classes of ichthyoarchaeological otoliths of *Micropogonias furnieri*, using R software (R Core Team 2012). (1) *A posteriori* test of body size medians versus shellmound localities. (2) *A posteriori* test of body size medians versus paleoenvironments where the shellmounds are located.

The results of ANOVA analyses (Kruskal-Wallis) on the median body sizes of whitemouth croaker show that, among the localities (Chi-squared = 1,042.3; df = 4, *p* values < 0.05), Beirada and Ponte do Girau shellmounds have significant similarities, and both are significantly different from other localities based on an *a posteriori* test (*p* values < 0.05). The comparative values of median body sizes from various environments and coastal geomorphologies (Chi-squared = 965.4, df = 2, *p* values < 0.05) reveal significant differences based on *a posteriori* tests (*p* values < 0.05) (Fig 5).

**Fig 6:**
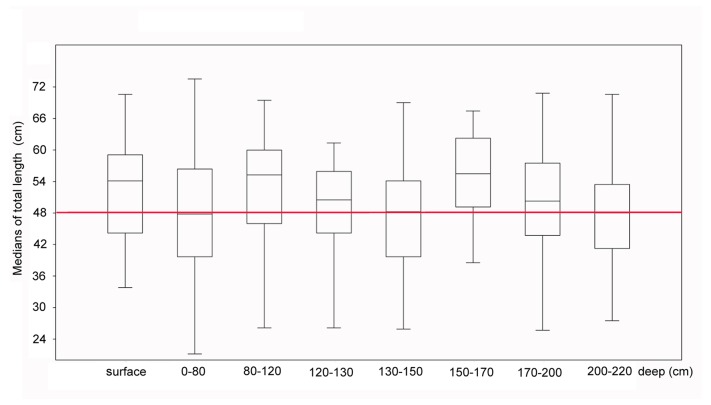
Comparative analyses of body size medians of whitemouth croaker from the multiple archaeological layers in the Manitiba shellmound in the Saquarema region. Note the differences among the layers, from the surface to deeper layers, and the trend of apparent cyclicity. The red line indicates the median of the total data set.

The overlap of age ranges between the sequence of archaeological layers (Fig 2) dismisses the multilayer interpretation of the body size distribution in a given shellmound, and we use the Manitiba shellmound as the best example of this. However, the median body sizes of whitemouth croakers in different layers of the Manitiba shellmound show different values and an apparent tendency toward cyclicity of medians from the archaeological surface layer toward deeper layers. This could be interpreted as the result of seasonal oscillations in the intensity of the seasonal marine coastal upwelling (Fig 6).

Individual drilled shark teeth included *Carcharodon carcharias*, *Carcharhinus altimus*, *C*. *leucas*, *C*. *plumbeus*, *Galeocerdo cuvier* and *Sphyrna mokarran* (Fig 7). The biodiversity of fish fauna records was illustrated based on individual diagnostic structures (i.e., otoliths, teeth or bones remains) from the recovered specimens (Figs 8–15).

**Fig 7:**
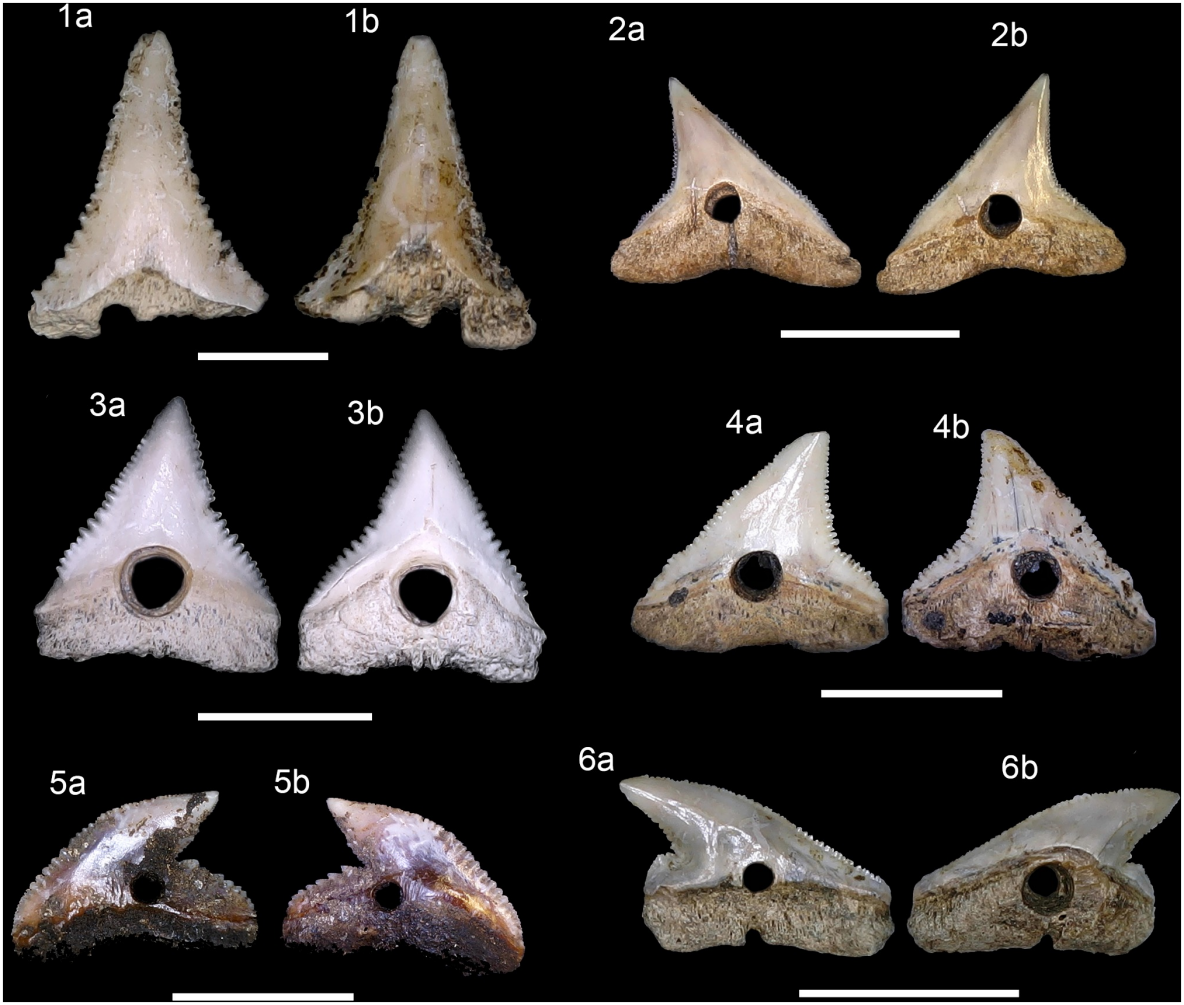
Drilled shark teeth. (1a-b) *Carcharodon carcharias*, lower tooth, Major, MNUFRJ-ZA-146. (2a-b) *Carcharhinus altimus*, upper tooth, Acaiá, MNUFRJ-ZA-868. (3a-b) *Carcharhinus* *leucas*, upper tooth, Ilha do Cabo Frio, MNUFRJ-ZA-869. (4a-b) *Carcharhinus* *plumbeus*, upper tooth, Caieira, MNUFRJ-ZA-97. (5a-b) *Galeocerdo cuvier*, indet. position tooth, Acaiá, MNUFRJ-ZA-870. (6a-b) *Sphyrna mokarran*, upper tooth, Algodão, MNUFRJ-ZA-54. Scale bar 1 cm.

**Fig 8:**
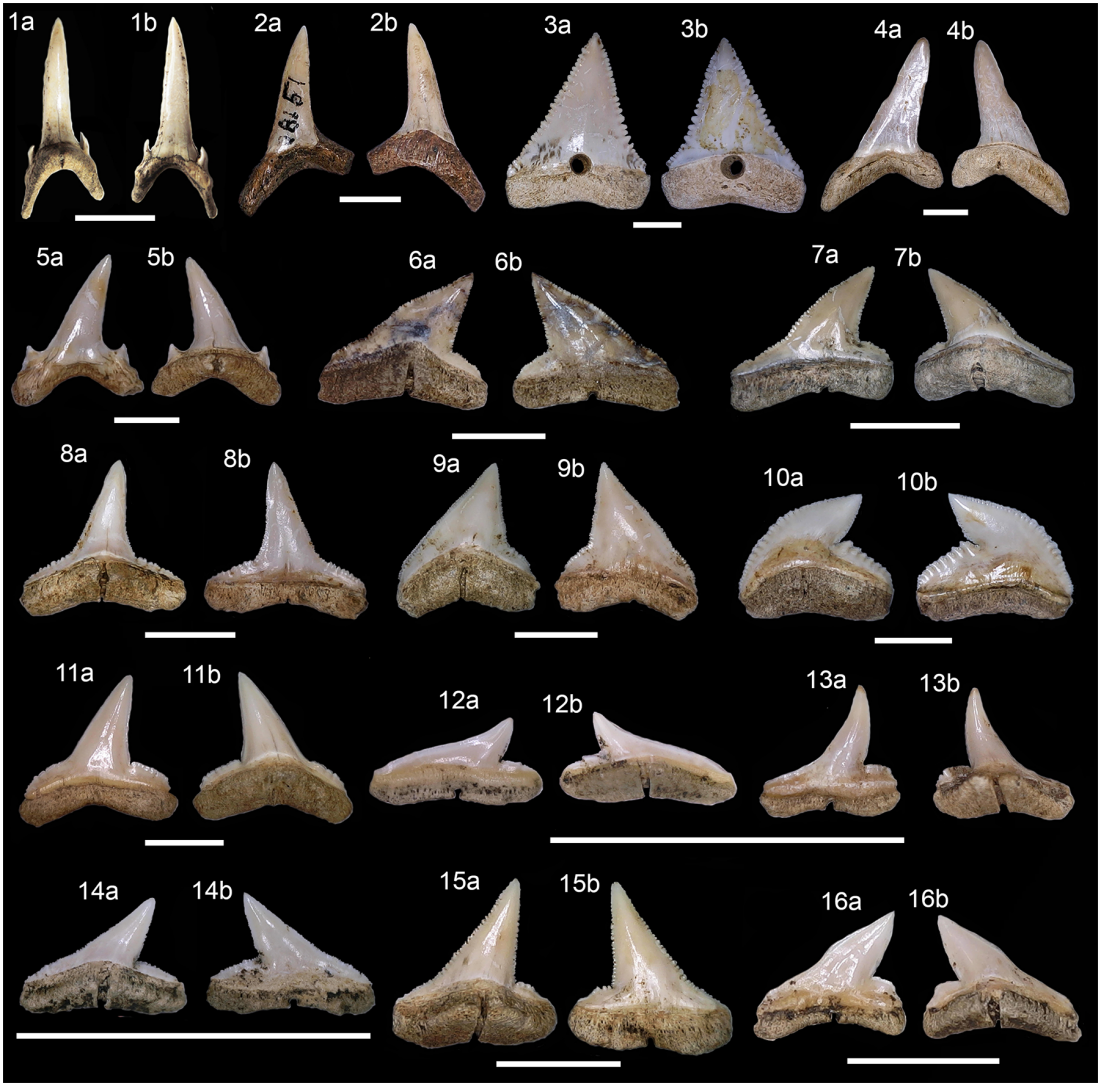
Shark teeth from Rio de Janeiro shellmounds. (1a-b) *Carcharias taurus*, upper tooth, Usiminas, MNUFRJ-ZA-565. (2a-b) *Alopias superciliosus*, upper tooth, Saquarema, MNUFRJ-ZA-Col.L.Kneip 28151. (3a-b) *Carcharodon carcharias*, upper tooth, Algodão, MNUFRJ-ZA-02. (4a-b) *Isurus oxyrinchus*, upper tooth, Algodão, MNUFRJ-ZA-21. (5a-b) *Lamna nasus*, lower tooth, Algodão, MNUFRJ-ZA-16. (6a-b) *Carcharhinus acronotus*, upper tooth, Algodão, MNUFRJ-ZA-01. (7a-b) *Sphyrna mokarran*, lower tooth, Major, MNUFRJ-ZA-149. (8a-b) *Carcharhinus limbatus*, upper tooth, Algodão, MNUFRJ-ZA-05. (9a-b) *Carcharhinus plumbeus*, upper tooth, Algodão, MNUFRJ-ZA-44. (10a-b) *Galeocerdo cuvier*, tooth, Algodão, MNUFRJ-ZA-17. (11a-b) *Negaprion brevirostris*, upper tooth, Algodão, MNUFRJ-ZA-25. (12a-b) *Rhizoprionodon lalandii*, lower tooth, Bigode, MNUFRJ-ZA-87, and (13a-b) upper tooth, Algodão, MNUFRJ-ZA-70. (14a-b) *Rhizoprionodon porosus*, upper tooth, Bigode, MNUFRJ-ZA-88. (15a-b) *Sphyrna mokarran*, upper tooth, Caieira II, MNUFRJ-ZA-99. (16a-b) *Sphyrna zygaena*, lower tooth, Major, MNUFRJ-ZA-161. Scale bar: 1 cm. Views: labial (1b, 2a-5a, 6b, 7a, 8-10b, 11-13a, 14-15b, and 16a), lingual (1a, 2-5b, 6a, 7b, 8-10a, 11-13b, 14-15a, and 16b).

**Fig 9:**
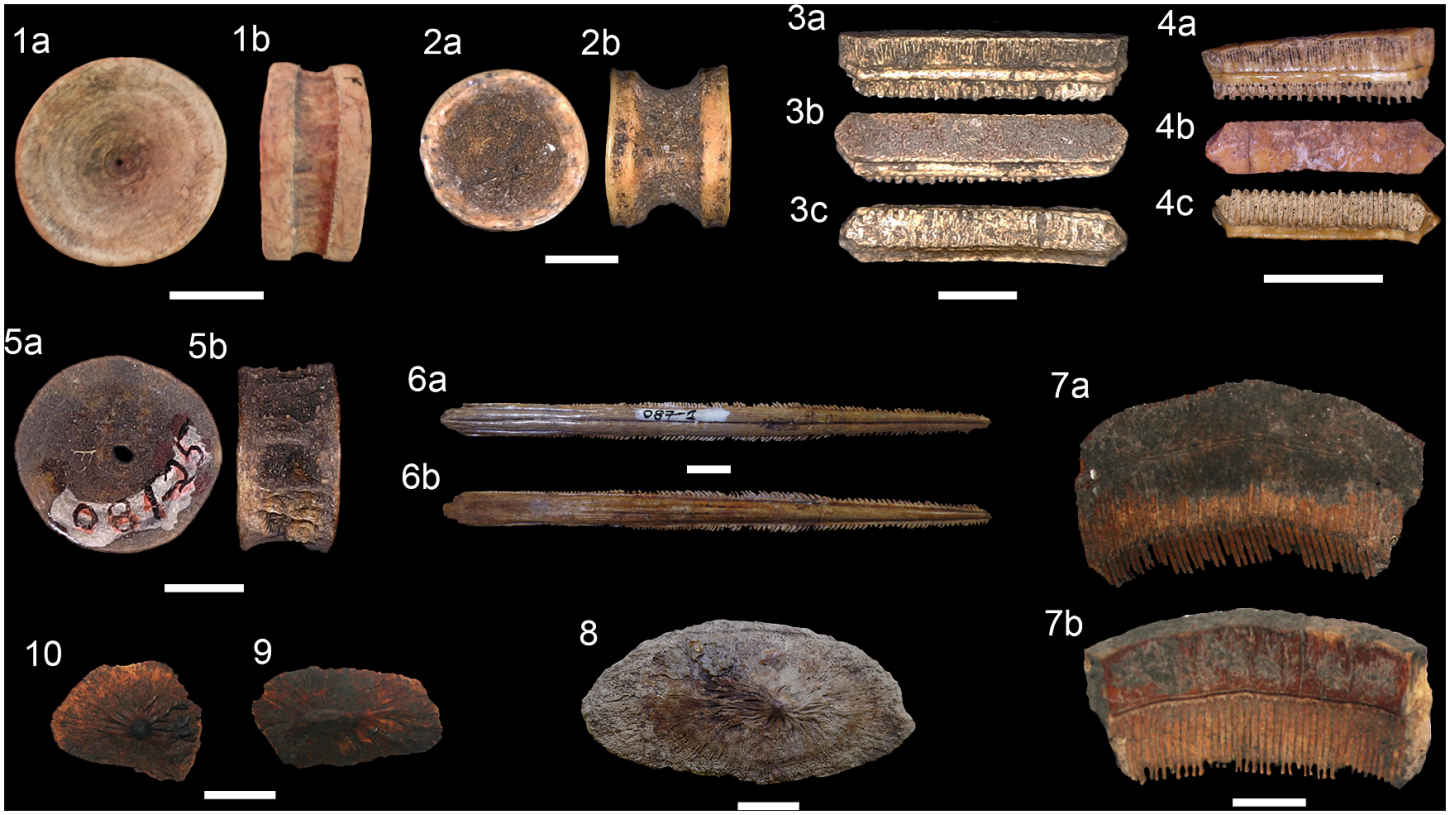
Ray teeth, vertebrae and dermal denticle from Rio de Janeiro shellmounds. (1a-b) *Pristis* sp., vertebrae, Usiminas, MNUFRJ-ZA-597 and, (2a-b) Algodão, MNUFRJ-ZA-624. (3a-c) *Rhinoptera* sp., tooth, Algodão, MNUFRJ-ZA-498, and (4a-c) Acaiá, MNUFRJ-ZA-708. (5a-b) Ray indet., vertebrae, Saquarema, MNUFRJ-ZA-Col.L.Kneip-08125. (6a-b) *Dasyatis centroura*, caudal spine, Saquarema, MNUFRJ-ZA-Col.L.Kneip-087-1. (7a-b) *Aetobatus narinari*, lower plate fragment, Usiminas, MNUFRJ-ZA-424. (8–10) Dasyatidae indet., dermal denticle, Usiminas, MNUFRJ-ZA-407. Scale bar: 1 cm.

**Fig 10:**
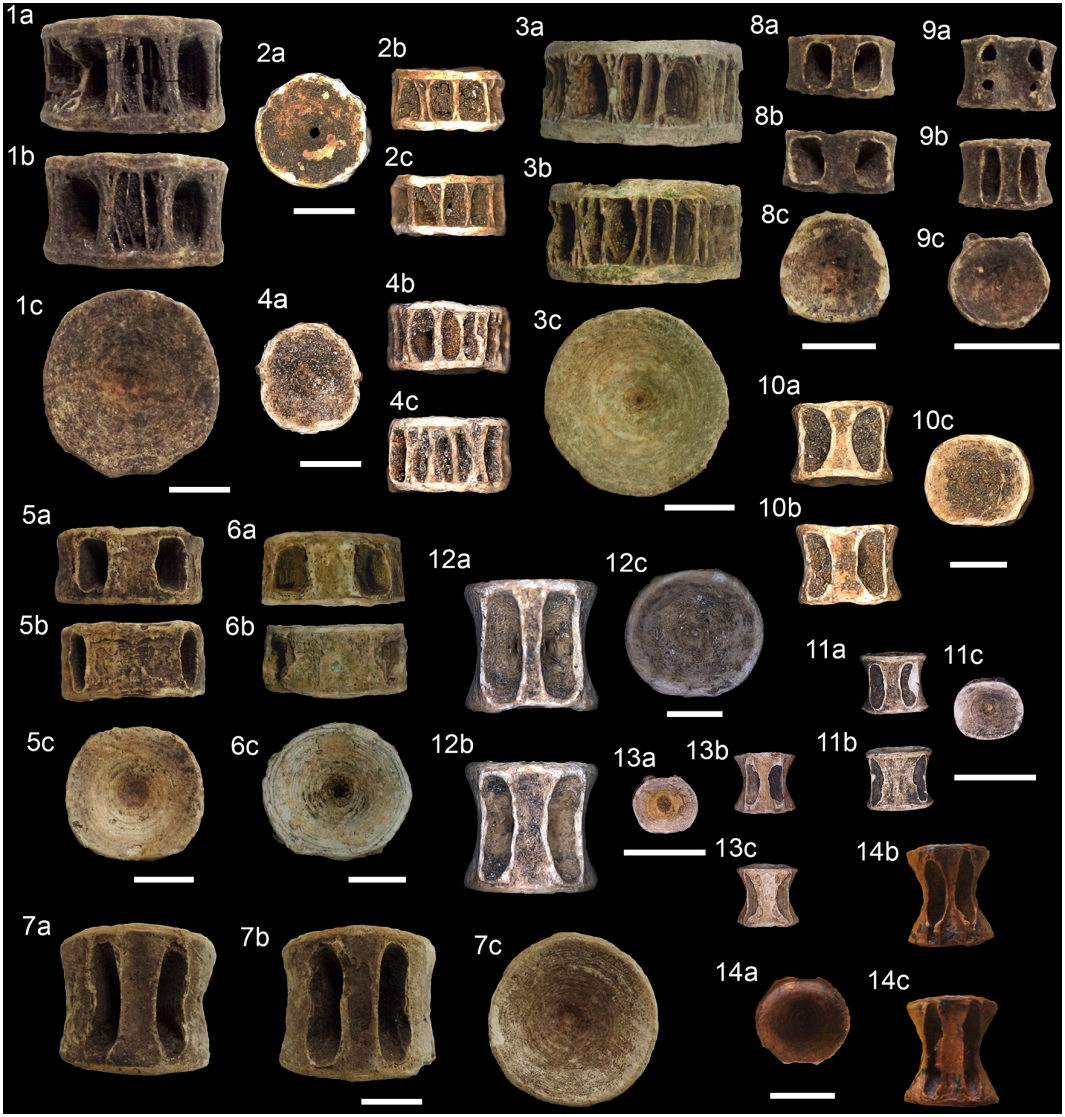
Shark and ray vertebrae from Rio de Janeiro shellmounds. (1a-c) *Carcharias taurus*, vertebrae, Usiminas, MNUFRJ-ZA-570. (2a-c) *Carcharodon carcharias*, vertebrae, Algodão, MNUFRJ-ZA-625, and (3a-b) Beirada, MNUFRJ-ZA-576. (4a-c) *Carcharias taurus*, vertebrae, Algodão, MNUFRJ-ZA-621. (5a-c) *Carcharhinus brevipinna*, anterior vertebrae, Usiminas, MNUFRJ-ZA-567, (6a-c) anterior vertebrae, Beirada, MNUFRJ-ZA-575, and (7a-c) posterior vertebrae, Usiminas, MNUFRJ-ZA-568. (8a-c) *Carcharhinus* sp., vertebrae, Usiminas, MNUFRJ-ZA-569. (9a-c) *Rhizoprionodon* sp., vertebrae, Usiminas, MNUFRJ-ZA-571. (10a-c) *Carcharhinus* sp., vertebrae, Algodão, MNUFRJ-ZA-620, (11a-c) Acaiá, MNUFRJ-ZA-715, and (12a-c) Algodão, MNUFRJ-ZA-623. (13a-c) *Sphyrna* sp., vertebrae, Usiminas, MNUFRJ-ZA-572, and (14a-c) Camboinhas, MNUFRJ-ZA-853. Scale bar: 1 cm.

**Fig 11:**
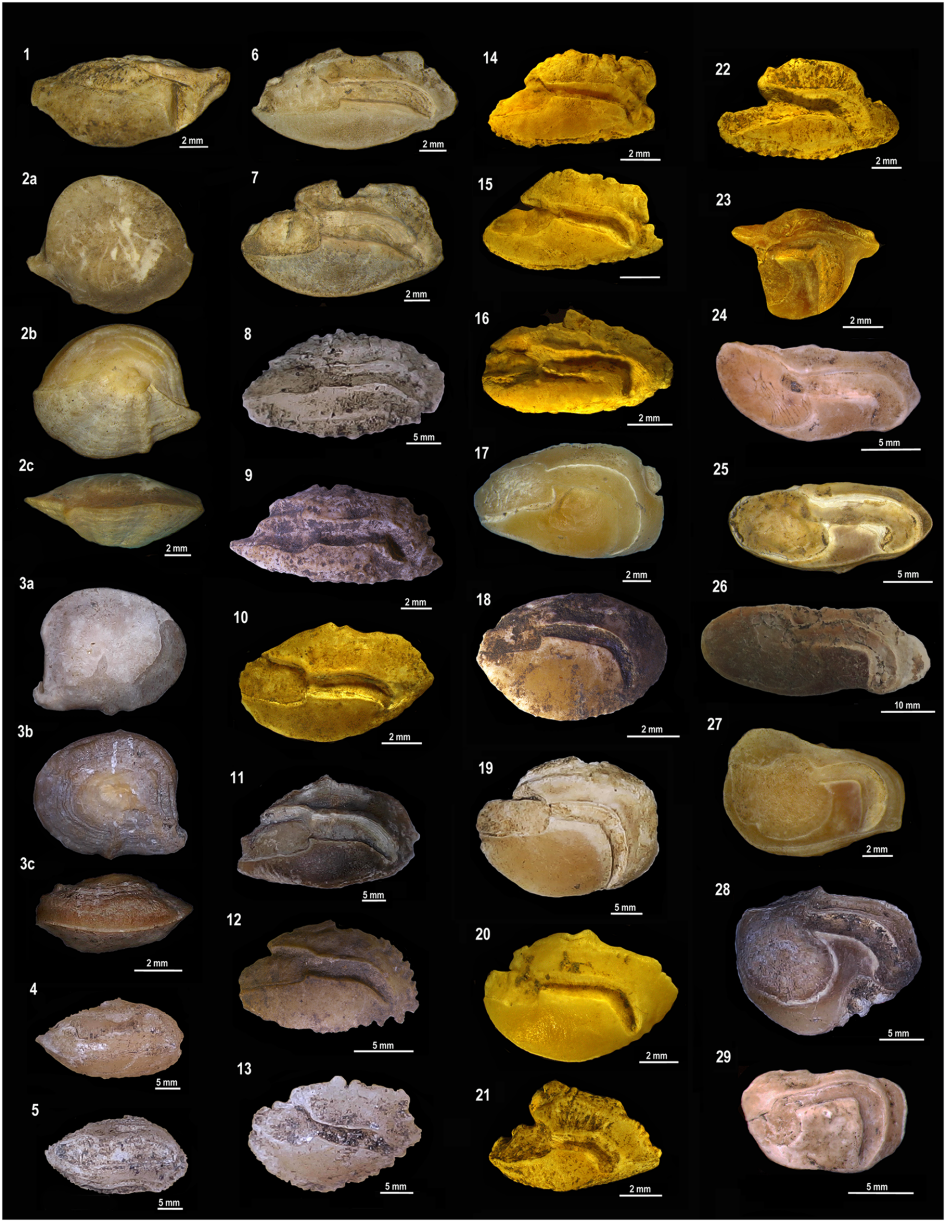
Teleostean otoliths from the Rio de Janeiro shellmounds. (1) *Albula nemoptera*, Algodão, MNUFRJ-ZA-190. (2a-c) *Bagre marinus*, Algodão, MNUFRJ-ZA-196. (3a-c) *Genidens genidens*, Camboinhas, MNUFRJ-ZA-845. (4) *Hemiramphus* sp., Caieira II, MNUFRJ-ZA-316, and (5) Caieira II, MNUFRJ-ZA-317. (6) *Centropomus ensiferus*, Algodão, MNUFRJ-ZA-206. (7) *Centropomus undecimalis*, Algodão, MNUFRJ-ZA-205. (8) *Epinephelus* sp., Major, MNUFRJ-ZA-269. (9) *Epinephelus marginatus*, Usiminas, MNUFRJ-ZA-521. (10) *Lutjanus synagris*, Algodão, MNUFRJ-ZA-228. (11) *Lutjanus* sp., Major, MNUFRJ-ZA-279. (12) *Ocyurus chrysurus*, Usiminas, MNUFRJ-ZA-519. (13) *Diapterus rhombeus*, Caieira II, MNUFRJ-ZA-306. (14) *Diplodus* sp., Algodão, MNUFRJ-ZA-215, and (15) Algodão, MNUFRJ-ZA-217. (16) *Gerres cinereus*, Algodão, MNUFRJ-ZA-223. (17) *Anisostremus virginicus*, Algodão, MNUFRJ-ZA-192. (18) *Haemulon aurolineatum*, Usiminas, MNUFRJ-ZA-517. (19) *Haemulon sciurus*, Algodão, MNUFRJ-ZA-184). (20) *Haemulon steindachneri*, Algodão, MNUFRJ-ZA-220. (21) *Archosargus rhomboidalis*, Algodão, MNUFRJ-ZA-194. (22) *Archosargus* sp., Algodão, MNUFRJ-ZA-193. (23) *Bairdiella ronchus*, Algodão, MNUFRJ-ZA-203. (24) *Cynoscion jamaicensis*, Acaiá, MNUFRJ-ZA-876. (25) *Cynoscion microlepidotus*, Algodão, MNUFRJ-ZA-213. (26) *Cynoscion acoupa*, Manitiba, MNUFRJ-ZA-559. (27) *Larimus breviceps*, Algodão, MNUFRJ-ZA-226. (28) *Micropogonias furnieri*, Algodão, MNUFRJ-ZA-232. (29) *Pareques acuminatus*, Manitiba, MNUFRJ-ZA-875.

**Fig 12:**
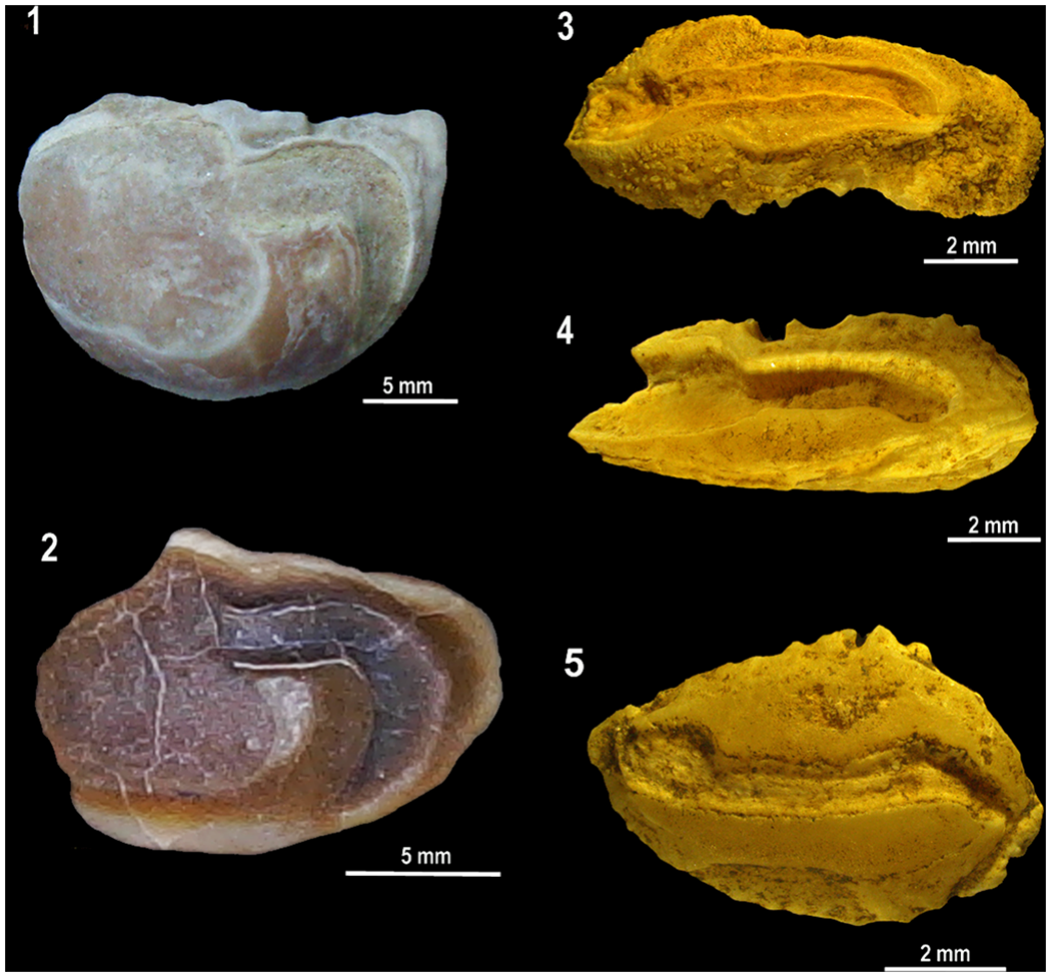
Teleostean otoliths from the Rio de Janeiro shellmounds. (1) *Pogonias cromis*, Ponte do Girau, MNUFRJ-ZA-562. (2) *Umbrina coroides*, Ilha do Cabo Frio, MNUFRJ-ZA-874. (3) *Mugil liza*, Algodão, MNUFRJ-ZA-233. (4) *Sphyraena guachancho*, Algodão, MNUFRJ-ZA-236. (5) *Hyporhamphus unifasciatus*, Algodão, MNUFRJ-ZA-2230.

**Fig 13:**
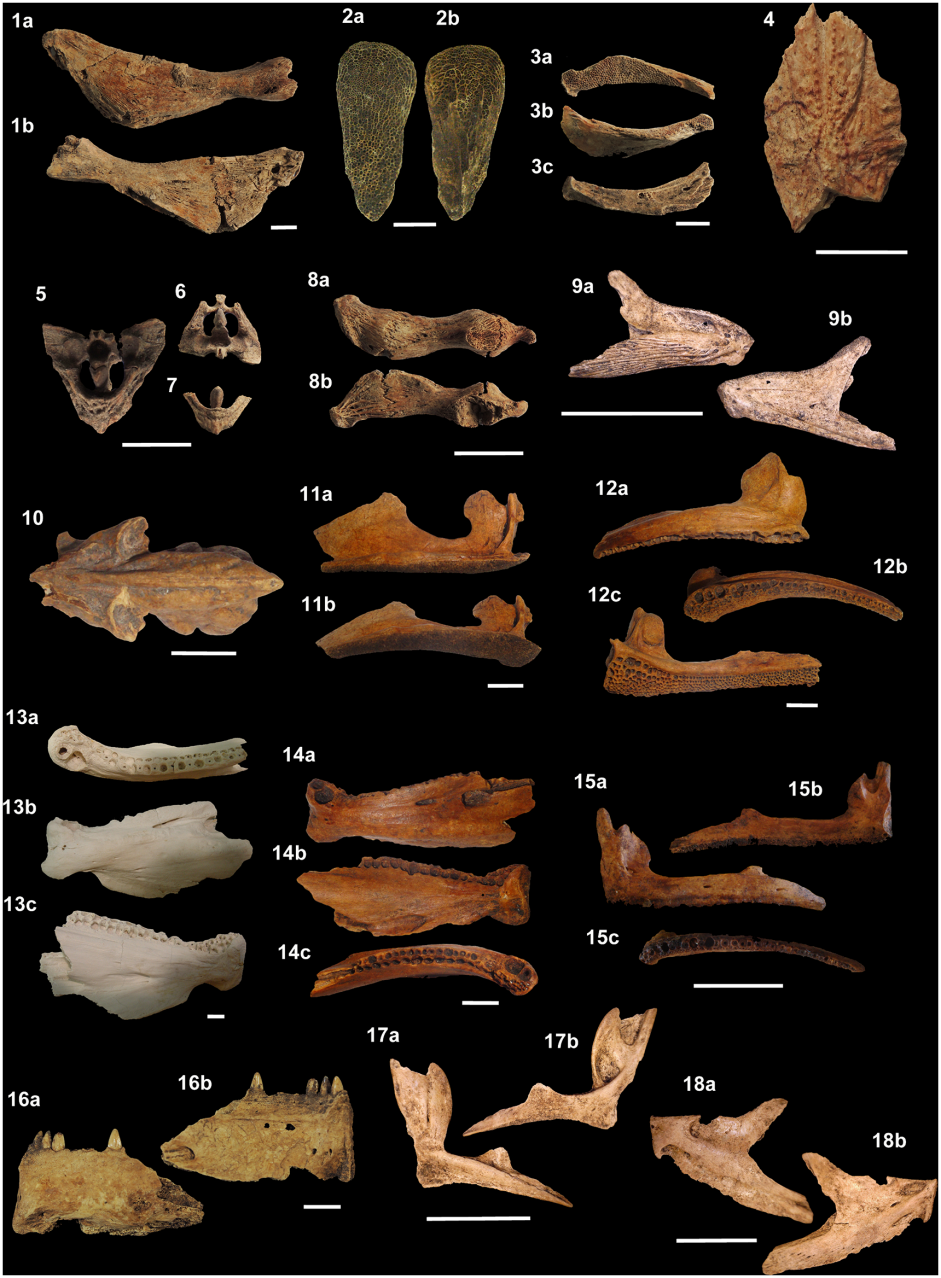
Teleostean skeletal remains from the Rio de Janeiro shellmounds. (1a-b) *Aspistor* sp., hyoid bar, Manitiba, MNUFRJ-ZA-537. (2a-b) *Bagre marinus*, frontal, Algodão, MNUFRJ-ZA-500. (3a-c) *Cathorops* sp., dentary, Manitiba, MNUFRJ-ZA-542. (4) *Genidens barbus*, supraoccipital, Manitiba, MNUFRJ-ZA-538. (5–7) *Genidens* sp., nucal plate, Manitiba, MNUFRJ-ZA-554, and (8a-b) hyoid bar, Manitiba, MNUFRJ-ZA-534. (9a-b) *Sargocentrum* sp., dentary, Acaiá, MNUFRJ-ZA-675. (10) *Centropomus parallelus*, supraoccipital, Usiminas, MNUFRJ-ZA-387. (11a-b) *Centropomus undecimalis*, premaxillary, Usiminas, MNUFRJ-ZA-439. (12a-c) *Epinephelus* sp., premaxillary, Usiminas, MNUFRJ-ZA-444, and (13a-c) dentary, Ilha do Cabo Frio, MNUFRJ-ZA-871. (14a-c) *Mycteroperca* sp., dentary, Usiminas, MNUFRJ-ZA-392. (15a-c) *Caranx* sp., premaxillary, Camboinhas, MNUFRJ-ZA-851, and (16a-b) dentary, Acaiá, MNUFRJ-ZA-744. (17a-b) *Selene vomer*, premaxillary, Acaiá, MNUFRJ-ZA-663, and (18a-b) dentary, Acaiá, MNUFRJ-ZA-667. Scale bar 1 cm.

**Fig 14:**
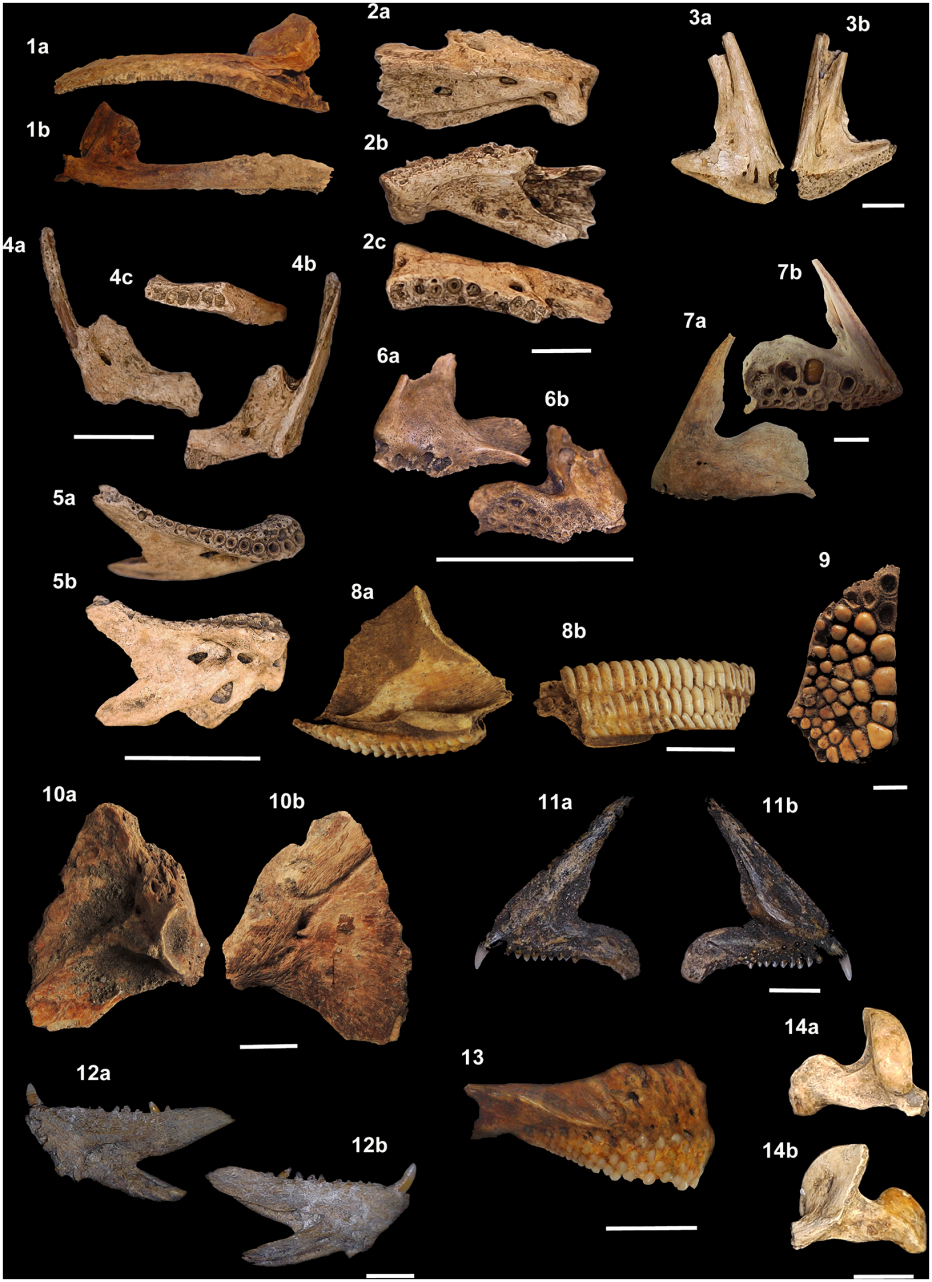
Teleostean skeletal remains from the Rio de Janeiro shellmounds. (1a-b) *Oligoplites saurus*, premaxillary, Usiminas, MNUFRJ-ZA-438. (2a-c) *Lutjanus* sp., dentary, Algodão, MNUFRJ-ZA-858. (3a-b) Gerreidae indet., premaxillary, Algodão, MNUFRJ-ZA-485. (4a-c) *Anisostremus* sp., premaxillary, Algodão, MNUFRJ-ZA-859, and (5a-b) dentary, Algodão, MNUFRJ-ZA-692. (6a-b) *Diplodus* sp., premaxillary, Acaiá, MNUFRJ-ZA-682. (7a-b) *Archosargus* sp., premaxillary, Ilha do Cabo Frio, MNUFRJ-ZA-873. (8a-b) *Sparisoma* sp., upper pharyngeal tooth plate, Acaiá, MNUFRJ-ZA-730. (9) *Pogonias cromis*, pharyngeal tooth, Manitiba, MNUFRJ-ZA-552. (10a-b) *Mugil* sp., opercle, Manitiba, MNUFRJ-ZA-550. (11a-b) *Bodianus rufus*, premaxillary, Acaiá, MNUFRJ-ZA-828, and (12a-b) dentary, Ilha do Cabo Frio, MNUFRJ-ZA-872. (13) *Sparisoma* sp., premaxillary, Usiminas, MNUFRJ-ZA-436. (14a-b) *Scarus* sp., maxillary, Algodão, MNUFRJ-ZA-993. Scale bar 1 cm.

**Fig 15:**
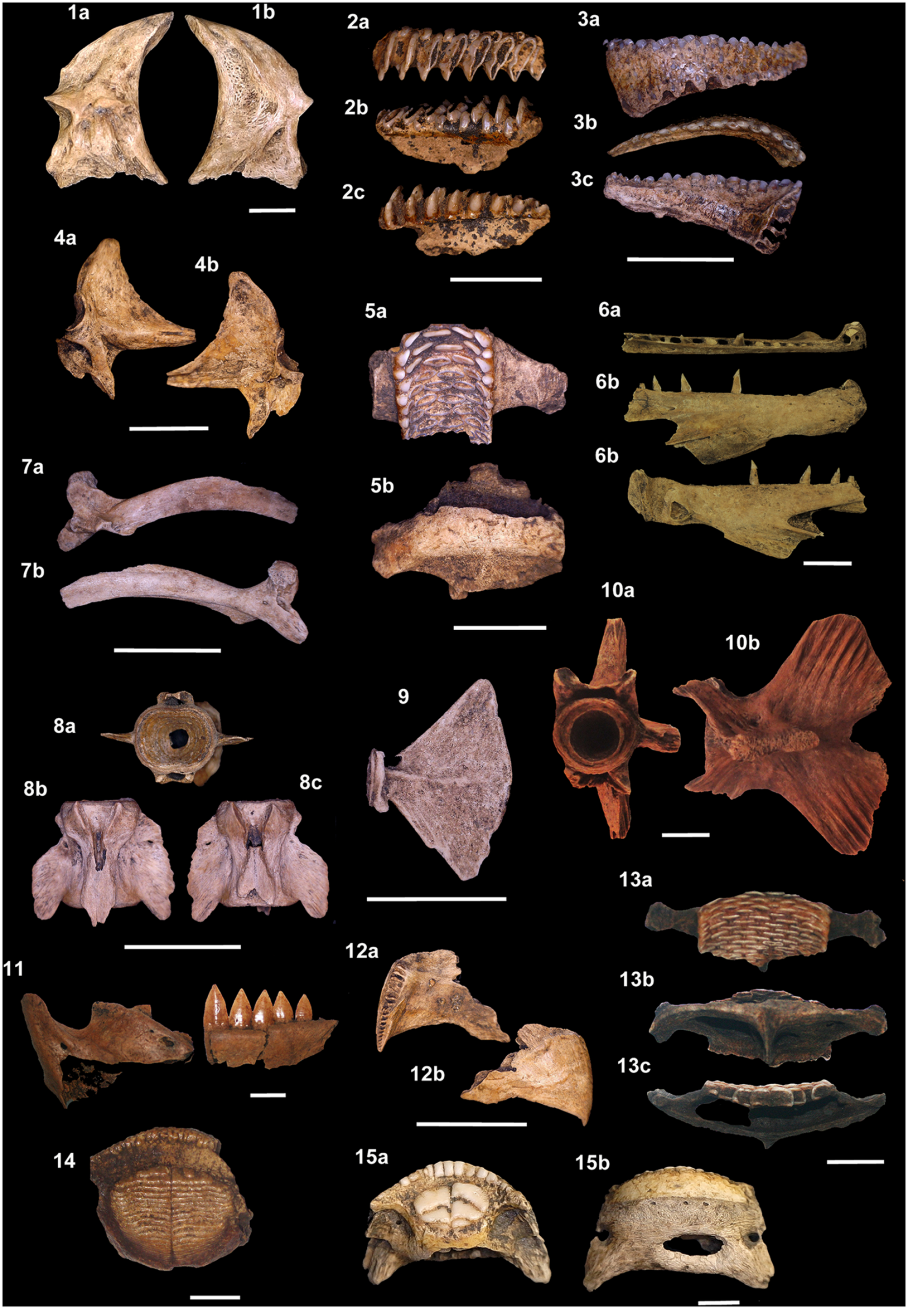
Teleostean skeletal remains from the Rio de Janeiro shellmounds. (1a-b) *Scarus* sp., angulo-articular, Algodão, MNUFRJ-ZA-496, and (2a-c) pharyngeal tooth, Acaiá, MNUFRJ-ZA-724. (3a-c) *Sparisoma* sp., dentary, Acaiá, MNUFRJ-ZA-720, and (4a-b) angulo-articular, Acaiá, MNUFRJ-ZA-666. (5a-b) *Scarus* sp., lower pharyngeal tooth plate, Acaiá, MNUFRJ-ZA-674. (6a-c) *Trichiurus lepturus*, dentary, Acaiá, MNUFRJ-ZA-746. (7a-b) *Katsuwonus pelamis*, maxillary, Acaiá, MNUFRJ-ZA-705, and (8a-c) vertebrae, Acaiá, MNUFRJ-ZA-710. (9) *Scomberomus* sp., hypural complex, Acaiá, MNUFRJ-ZA-701. (10a-b) *Istiophorus albicans*, hypural, Usiminas, MNUFRJ-ZA-471. (11) *Sphyraena barracuda*, dentary, Usiminas, MNUFRJ-ZA-395. (12a-b) *Lagocephalus laevigatus*, premaxillary, Acaiá, MNUFRJ-ZA-679. (13a-c) *Sparisoma* sp., lower pharyngeal tooth plate, Usiminas, MNUFRJ-ZA-428. (14) *Diodon* sp., tooth plate, Usiminas, MNUFRJ-ZA-429. (15a-b) *Chilomycterus spinosus*, dentary, Algodão, MNUFRJ-ZA-487. Scale bar 1 cm.

## Discussion

We recognized 97 fish species from the Rio de Janeiro shellmounds based on detailed anatomic analysis of diagnostic structures. However, some taxonomic records (i.e., at least 44 taxa) that were previously cited in technical reports and unpublished theses about the Rio de Janeiro shellmounds could not be located in the MN-UFRJ repository for identification. These unexamined species are not under institutional catalogue records, and their descriptions or illustrations are not available. We, therefore, choose to exclude those ‘specimens’ from our analysis.

Population structures from shellmounds suggest that the estimated body length of coastal species (e.g., carcharhinids) follows a common pattern of juvenile and adult body length. Moreover, very small teeth and vertebrae of lamnids and sphyrnids collected from the shellmounds of Angra dos Reis and Cabo Frio Island indicate the possible presence of pregnant females, neonates and juveniles in a protected coastal area. Similar evidence is provided by large and very small vertebrae of sawfishes, *Pristis* spp. (Fig 9).

The *Carcharhinus brevipinna* size estimation is in agreement with the common length of contemporary specimens (i.e., 250 cm TL [71]), and the species is classified as a threatened species [103]. The estimated sizes of *Carcharias taurus* are two times smaller than those of their living counterpart (i.e., 250 cm TL [71]), which is classified as a vulnerable species [104]. Juvenile sizes of *Carcharhinus* species coincide with the most common length of the living counterpart. *Sphyrna* spp. size is nine times smaller than the common length estimation of the living counterpart and could be represented here by neonate specimens [in comparison with the adults body size (i.e., 370 cm TL [88])]. *Sphyrna* species are currently classified as an endangered species [105]. The size of *Carcharodon carcharias* is three times smaller than the common body length of their living counterpart (i.e., 541 cm TL for males and 594 cm for females [88]), which is classified as a vulnerable species [106]. The presence of small vertebrae (and some teeth) is suggestive of neonates of Lamnidae, Carcharhinidae, Sphyrnidae and Pristidae species and provides irrefutable support for the presence of pregnant females and juveniles in these nursery areas.

There are three criteria to identify an area as a nursery [107]: **(1)**, an area with a high frequency of sharks; **(2)**, an area to which shark species have a tendency to remain or return for extended periods; **(3)**, an area or habitat that is repeatedly used across years. Sharks’ philopatry [108] and the evidence of residence and the site fidelity suggest that oceanic species preferentially return to their exact birthplaces (i.e., natal philopatry) or birth regions (i.e., regional philopatry) for either parturition or mating even though they make long distance movements that would allow them to breed elsewhere [109]. Modern philopatric are represented by Holocene species in the Brazilian shellmounds, and include *Carcharias taurus*, *Carcharodon carcharias*, *Carcharhinus leucas*, *C*. *limbatus*, *Galeocerdo cuvier* and *Negaprion brevirostris* [109].

Special remarks are made regarding the presence of cosmopolitan sharks with long-distance oceanic migration, such as *G*. *cuvier* and *C*. *carcharias*. The tiger shark, *G*. *cuvier*, spends the majority of its time in the upper 50 m of water and is recorded to migrate approximately 1,100 to 1,800 km [110,111,112]. The white shark, *C*. *carcharias*, during its ‘patrolling’ behavior, mostly swam in depths between 5 and 50 m and during migration, swam almost exclusively at the surface [113]. The long distance oceanic migration of this shark is record to be approximately 4,000 km between the Pacific coast of California and Mexico to the Hawaiian islands [113,114]. Both tiger and white sharks were represented in the shellmounds by perforated teeth, some of them recovered as pendant necklaces associated with human burials and bones [115]. Moreover, modern shark nursery areas were recognized in Brazil [116,117].

Estimated teleostean size class ranges in selected shellmounds, based on 5,532 otoliths of *Micropogonias furnieri*, showed a long history of fishery exploitation (ca 5,595 cal BP until today). The frequency distribution of whitemouth croaker, *M*. *furnieri*, body sizes based on otoliths from the Ponte do Girau, Algodão, Beirada, Manitiba and Camboinhas shellmounds, and the body size (TL) estimates from modern fisheries (i.e., artisanal and semi-industrial) in the Itaipu and Angra dos Reis regions, Rio de Janeiro State (Fig 4), suggest two modal distributions of body size frequencies, with overlap between prehistoric and modern fisheries. In comparison to the size estimated based on otoliths of *M*. *furnieri* from the shellmounds, a probabilistic tendency of a reduction in body size of 28% in modern catches may be attributed to overexploitation.

Despite *M*. *furnieri* being a species with medium resilience to fishing pressure (i.e., criteria based on values of selected life-history parameters: high fertility and high body growth parameter, K [118]), the regional assessments of current stocks indicate overexploitation [89,90]. Results indicate that body size medians between shellmounds and related environment/coastal geomorphology were significantly different (Fig 5). These differences could be interpreted as a consequence of seasonal variations of fishery areas and the intrinsic life history of the species.

The prehistoric fisheries and time series records along archaeostratigraphic sections vary from a single to seven layers based on the chronological sequences of settlements studied here, while the calibrated radiocarbon models, based on fish otoliths and shell samples by layer, show close or overlapping age probability distributions of individual shellmounds [52,53,55]. Consequently, the shallow profile of individual shellmounds studied here from Rio de Janeiro State could be treated arbitrarily as a single unit for the purpose of a fisheries analysis.

The results, in terms of richness, point toward an early stage of overexploitation of medium- to large-sized, vulnerable fish species, characterized by late maturity, slow growth, low reproductive rates, longevity, spawning aggregations and often ovoviviparous and/or viviparous life histories [119,120,121]. Groupers also exhibit slow growth, low reproductive rates and increased longevity, and reproductive adults leave shallow water habitats and move to deep waters after spawning [122,123]. Our results suggest that prehistoric fishing pressure on coastal areas was sufficient to cause the initial phase of population declines [58,124,125].

Prehistoric fishery methods were able to catch medium to large sharks, skipjack tunas, sailfish and groupers and could have included seined or floating gillnets, spears and long-lines. However, more resilient demersal species did not decline as drastically. Therefore, it is plausible that seasonal fisheries that used beach seines during high upwelling productivity contributed to massive catches of groundfish schools. Other small species could be caught by hooks or traps.

Sawfishes were present during prehistoric times in southeastern Brazil [126]; however, nowadays, these species are a nearly extinct taxon [127], and the last record of the great white shark was in the mid 80’s [128]. *Carcharias taurus* is a common shark caught in summer in small numbers in artisanal gillnet fisheries on the Rio de Janeiro coast [128]. Nothing is known about the porbeagle shark, *Lamna nasus*, in the Brazilian region, a rare shark caught in longlines from the 60’s to the 90’s [129]. This species is distributed from southern Brazil and Uruguay to Argentina [130].

Diverse evidence of archaeological artifacts of gorges, bones and shell fishhooks (including an early fishhook, dated to 42,000 yr BP [131]) and fishing lines (from native fiber plants or human hair) used for inshore or pelagic fisheries was recorded worldwide [15,132,133]. The presence of projectile points in archaeological sites in California, USA (~ 12,200 to 11,200 yr BP) associated with marine and aquatic faunal remains is common [134]. Moreover, the only known evidence of fishery tools from Brazilian archaeological sites are manufactured bony spear-tips [30], without clear evidence of possible techniques for massive catches or refined manufacture of hooks used for pelagic or large demersal fishes (S4 Appendix).

Shark teeth are culturally significant in the worldview of indigenous mythology, and numerous archaeological burials of human skeletons in Brazilian shellmounds were decorated with necklaces made with drilled shark teeth [115]. Drilled shark teeth are also present in Argentinian and Uruguayan middens [135]. Individually, drilled shark teeth are here represented by *Carcharodon carcharias*, *Galeocerdo cuvier*, *Carcharhinus altimus*, *C*. *leucas*, *C*. *plumbeus* and *Sphyrna mokarran* (Fig 7). However, another possibility is the use of shark teeth for tool manufacturing, e.g., affixed to pieces of wood with vegetal fibers, similar to Polynesian artifacts [127], or as points of arrows.

The analysis of the ichthyofauna shows significant differences between expected fish assemblages (i.e., based on modern occurrences in the coastal area) and the record of observed species in shellmounds (Table 2, Figs 7–15). A high diversity of fish species in the shellmounds reveals the fishery and coastal navigation skills of ancient fishermen and the high importance of coastal fisheries for those prehistoric communities. Such prehistoric fishery activities on vulnerable species and special nursery areas could correspond to the beginning of fish stock depletion along the southeastern Brazilian coast. Overexploitation of such coastal fisheries became unequivocally intense during colonial times resulting in the near collapse of natural fish populations, especially affecting large-sized species such as sharks and giant groupers.

However, in terms of natural resources, we assume that eight biases might have affected the ichthyoarchaeological samples under study: **(1)**, selective targeting of certain species (i.e., unpalatable taste, poisonous fishes, small sizes, etc.); **(2)**, the inaccessibility of certain available species (e.g., limitations of employed fishing techniques or presence of adverse marine environmental conditions); **(3)**, minimal potential preservation of some species (i.e., some fishes such as sardines could be consumed entirely); **(4)**, non-uniform employment of archaeological sieving techniques (i.e., the use of large mesh sizes and loss of small stingray teeth such as those of *Dasyatis*, *Gymnura*, or *Mobula* species and otoliths, like those of syngnathids, cynoglossids, achirids, atherinids, bleniids and gobiids); **(5)**, archaeological priority (i.e., ichthyoarchaeological remains such as bones, teeth and otoliths could be considered of secondary importance during field activities, except when they exhibit holes, cutting or intentional abrasion); **(6)**, early overexploitation and fish stock depletion (i.e., large and diverse shark species and rocky reef fish are well represented only in the Angra dos Reis shellmounds); **(7)**, the presence of non-diagnostic or broken bones or eroded otoliths (i.e., unclassified species); and **(8)**, missing specimens.

## Conclusions

Prehistoric fishery activity along the Rio de Janeiro coast under the influence of coastal marine upwellings was characterized by massive catches of demersal finfish that inhabit sandy and coastal marine lagoons (e.g., *Micropogonias furnieri*), rocky reef fishes caught near islands and islets (e.g., *Epinephelus morio*), and pelagic fishes caught near rocky cliffs and islands (e.g., *Istiophorus albicans*). Shark fisheries could have been located in nursery areas of protected rocky cliff bays in Arraial do Cabo, Cabo Frio Island, Angra dos Reis and Ilha Grande.

Prehistoric records of high elasmobranch diversity in the Ribeira Bay provide clear evidence for the exploitation of natural populations of sharks and rays since pre-colonial times, especially of bigger species such as the porbeagle shark, *Lamna nasus*, the sand tiger shark, *Carcharias taurus*, the great white shark, *Carcharodon carcharias*, and sawfishes, *Pristis* sp. All these are vulnerable species that could have been rare, especially when taking into consideration that today they are rare or present in reduced numbers in the Ribeira Bay or in the adjacent Angra dos Reis region.

Hence, the results produced here should be addressed as a baseline reference of the ichthyodiversity during the prehistoric times, promoting further debate on the relationships established with past fishing activities, as well as changes in local and regional oceanographic systems.

## Supporting Information

S1 Appendix Distribution of shellmounds along the South American coast. (DOCX)

S2 Appendix Ichthyoarchaeological material. Ichthyological collection, Zooarchaeology, Museu Nacional, Universidade Federal do Rio de Janeiro (UFRJ)–curators: Maria Cristina Tenório, Tânia Lima.
(DOCX)

S3 Appendix Modern material. A. Ichthyological collection, Departamento de Biologia Animal e Vegetal—Instituto de Biologia, Universidade do Estado do Rio de Janeiro (UERJ)—curators: Ulisses Leite Gomes, Maisa da Cruz Lima, Cristina Paragó, Alexandra Pinto Quintans. B. Ichthyological collection, Otoliths, Departamento de Biologia Marinha—Instituto de Biologia, Universidade Federal Fluminense (UFF)–curator: Orangel Aguilera. C. Ichthyological collection, Dry skeletons, Departamento de Biologia Marinha—Instituto de Biologia, Universidade Federal Fluminense (UFF)–curators: Orangel Aguilera.
(DOC)

S4 Appendix The unknown prehistoric fishing. Artwork by Eduardo Agelvis.
(DOCX)
