# Supplementary material for: The Path towards Endangered Species: Prehistoric Fisheries in Southeastern Brazil
Source: PLoS One. 2016 Jun 29;11(6):e0154476. doi: 10.1371/journal.pone.0154476 (PMC4939631; doi:10.1371/journal.pone.0154476)
Supplement: S1 Appendix — (DOCX) [file pone.0154476.s001.docx]

**S1 Appendix. Distribution of shellmounds along the South American coast.**

**
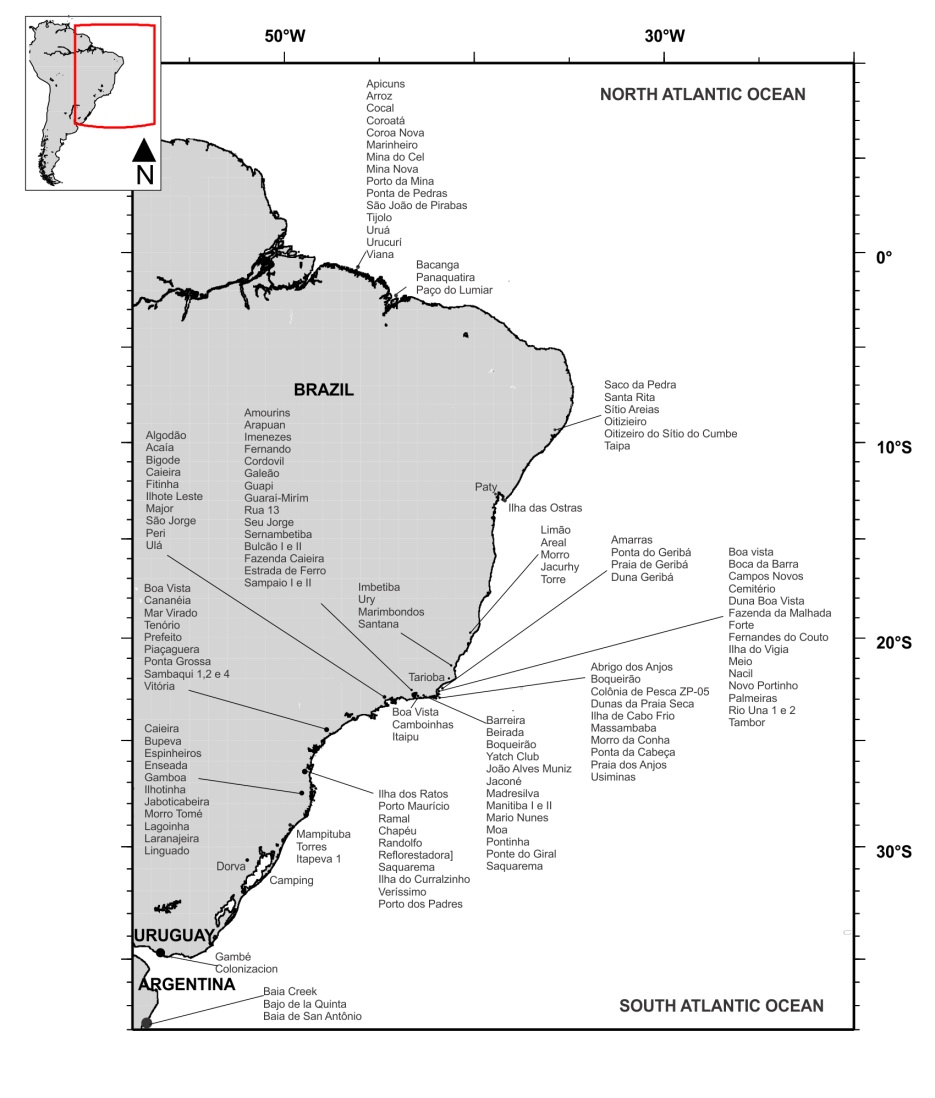
**

**Figure 1. Map of the distribution of shellmounds along the South American coast**. According to Etchevarne 2000; Duboi and Scartascini 2012; Beovidei and Martinezii 2014; Lima 1999/2000; Silva 2013; Silvana-Santana et al. 2013; Silveira and Schaan 2005; Souza et al. 2011; Wagner et al. 2011.

**References**

1. Etchevarne C. Ocupação Humana no nordeste brasileiro antes da colonização portuguesa. Rev USP, São Paulo, 2000; 44: 112-141.
2. Favier Dubois CM, Scartascini F. Intensive fishery scenarios on the North Patagonian coast (Río Negro, Argentina) during the Mid-Holocene. Quat Int, 2012; 256: 62-70.
3. Beovidei LL, Martinezii S. Concheros Arqueológicos en la Costa Uruguaya: Revisión y Perspectivas Archaeological Shell Middens in the Uruguayan Coast: Review and Perspectives. Rev Chil Antropol, 2014; 1:26-31.
4. Lima TA. Em busca dos frutos do mar: os pescadores- coletores do litoral centro-sul do Brasil. Rev USP, 1999/2000; 44:270- 327.
5. Silva DF. Análise da capitação de recursos da área do sambaqui Saco da Pedra, litoral sul do estado de Alagoas. M.sc. Thesis, Universidade Federal de Pernambuco. 2013. Available: http://repositorio.ufpe.br:8080/xmlui/handle/123456789/429.
6. Silva-Santana CC, Silva AC, Gasparino AC, Lima FS, Vieira NS, Santana JAB. Sambaqui do Paty ou um sítio multicomponencial para coleta de mariscos? Rev Eletrônica Lab Arqueol Paleontol da UEPB, 2013; 1-6.
7. Silveira MI. Schaan DP. Onde a Amazônia encontra o mar: estudando os sambaquis do Pará. Rev Arqueol, 2005; 18: 67-79.
8. Souza RCCL,; Lima TA, Silva EP. Conchas Marinhas de Sambaquis do Brasil. 1 st ed. Rio de Janeiro: Technical Books Editora, 2011.
9. Wagner G, Hilbert K, Bandeira D, Tenório MC, Okumura MM. Sambaquis (shell mounds) of the Brazilian coast Quat Int, 2001; 239:51-60. doi: 10.1016/j.quaint.2011.03.009.
