# Supplementary material for: The Path towards Endangered Species: Prehistoric Fisheries in Southeastern Brazil
Source: PLoS One. 2016 Jun 29;11(6):e0154476. doi: 10.1371/journal.pone.0154476 (PMC4939631; doi:10.1371/journal.pone.0154476)
Supplement: S2 Appendix — Ichthyological collection, Zooarchaeology, Museu Nacional, Universidade Federal do Rio de Janeiro (UFRJ)–curators: Maria Cristina Tenório, Tânia Lima. (DOCX) [file pone.0154476.s002.docx]

**S2 Appendix. Ichthyoarchaeological material.** Ichthyological collection, Zooarchaeology, Museu Nacional, Universidade Federal do Rio de Janeiro (UFRJ) – curators: Maria Cristina Tenório, Tânia Lima.

CHONDRICHTHYES

ODONTASPIDIDAE

*Carcharias taurus* (Usiminas, MNUFRJ-ZA-56, Usiminas, MNUFRJ-ZA-570, Algodão, MNUFRJ-ZA-621)

ALOPIDAE

*Alopias supersicliosus* (Saquarema, MNUFRJ-ZA-Col.L.Kneip 28151)

LAMNIDAE

*Carcharodon carcharias* (Major, MNUFRJ-ZA-146, Algodão, MNUFRJ-ZA-02, Algodão, MNUFRJ-ZA-625, Beirada, MNUFRJ-ZA-576); *Isurus oxyrinchus* (Algodão, MNUFRJ-ZA-21); *Lamna nasus* (Algodão, MNUFRJ-ZA-16)

CARCHARHINIDAE

*Carcharhinus acronotus* (Algodão, MNUFRJ-ZA-01); *Carcharhinus* *altimus* (Acaiá, MNUFRJ-ZA-868); *Carcharhinus brevipinna* (Usiminas, MNUFRJ-ZA-567, Beirada, MNUFRJ-ZA-575, Usiminas, MNUFRJ-ZA-568, Usiminas, MNUFRJ-ZA-573); *Carcharhinus leucas* (Ilha do Cabo Frio, MNUFRJ-ZA-869); *Carcharhinus limbatus* (Algodão, MNUFRJ-ZA-05); *Carcharhinus plumbeus* (Algodão, MNUFRJ-ZA-44, Caieira, MNUFRJ-ZA-97); *Carcharhinus priscus* (Usiminas, MNUFRJ-ZA-578)*; Carcharhinus* sp. (Usiminas, MNUFRJ-ZA-569, Algodão, MNUFRJ-ZA-620, Acaiá, MNUFRJ-ZA-715, Algodão, MNUFRJ-ZA-623); *Galeocerdo cuvier* (Acaiá, MNUFRJ-ZA-870, Algodão, MNUFRJ-ZA-17); *Negaprion brevirostris* (Algodão, MNUFRJ-ZA-25); *Rhyzoprionodon lalandii* (Bigode, MNUFRJ-ZA-87, Algodão, MNUFRJ-ZA-70); *Rhyzoprionodon porosus* (Bigode, MNUFRJ-ZA-88); *Rhizoprionodon* sp. (Usiminas, MNUFRJ-ZA-571)

SPHYRNIDAE

*Sphyrna mokarran* (Algodão, MNUFRJ-ZA-54, Major, MNUFRJ-ZA-149, Caieira II, MNUFRJ-ZA-99); *Sphyrna zygaena*, (Major, MNUFRJ-ZA-161); *Sphyrna* sp. (Usiminas, MNUFRJ-ZA-572, Camboinhas, MNUFRJ-ZA-853)

PRISTIDAE

*Pristis* sp. (Usiminas, MNUFRJ-ZA-597, Algodão, MNUFRJ-ZA-624)

DASYATIDAE

*Dasyatis centroura* (Saquarema, MNUFRJ-ZA-Col.L.Kneip-087-1); Dasyatidae (Usiminas, MNUFRJ-ZA-407)

MYLIOBATIDAE

*Aetobatus narinari* (Usiminas, MNUFRJ-ZA-424);); *Rhinoptera* sp. (Acaiá, MNUFRJ-ZA-708, Algodão, MNUFRJ-ZA-498, MNUFRJ-ZA-Col.L.Kneip-08125)

OSTEICHTHYES

ALBULIDAE

*Albula nemoptera* (Algodão, MNUFRJ-ZA-190)

ARIIDAE

*Aspistor* sp. (Manitiba, MNUFRJ-ZA-537); *Bagre bagre; Bagre marinus* (Algodão, MNUFRJ-ZA-196, MNUFRJ-ZA-500); *Cathorops* sp. (Manitiba, MNUFRJ-ZA-542); *Genidens genidens* (Camboinhas, MNUFRJ-ZA-845); *Genidens barbus* (Manitiba, MNUFRJ-ZA-538); *Genidens* sp. (Manitiba, MNUFRJ-ZA-554, MNUFRJ-ZA-534)

HOLOCENTRIDAE

*Sargocentrum* sp. (Acaiá, MNUFRJ-ZA-675)

HEMIRAMPHIDAE*Hemiramphus* sp. (Caieira II, MNUFRJ-ZA-316, Caieira II, MNUFRJ-ZA-317)

CENTROPOMIDAE

*Centropomus ensiferus* (Algodão, MNUFRJ-ZA-206); *Centropomus undecimalis* (Algodão, MNUFRJ-ZA-205, Usiminas, MNUFRJ-ZA-439); *Centropomus parallelus* (Usiminas, MNUFRJ-ZA-387)

SERRANIDAE

*Epinephelus* sp.(Major, MNUFRJ-ZA-269, Usiminas, MNUFRJ-ZA-444*,* Ilha do Cabo Frio, MNUFRJ-ZA-871); *Epinephelus marginatus* (Usiminas, MNUFRJ-ZA-521); *Mycteroperca* sp. (Usiminas, MNUFRJ-ZA-392)

CORYPHAENIDAE

*Coryphaena hippurus* (Acaiá, MNUFRJ-ZA-741)

CARANGIDAE

*Caranx hippos* (Usiminas, MNUFRJ-ZA-414, MNUFRJ-ZA-413, MNUFRJ-ZA-475, Niterói, MNUFRJ-ZA-843); *Caranx* sp. (Camboinhas, MNUFRJ-ZA-851, Acaiá, MNUFRJ-ZA-744); *Selene vomer* (Acaiá, MNUFRJ-ZA-663, Acaiá, MNUFRJ-ZA-667); *Oligoplites saurus* (Usiminas, MNUFRJ-ZA-438); *Selar crumenophthalmus* (Acaiá, MNUFRJ-ZA-662)

LUTJANIDAE

*Lutjanus synagris* (Algodão, MNUFRJ-ZA-228); *Lutjanus* sp. (Major, MNUFRJ-ZA-279, Algodão, MNUFRJ-ZA-858); *Ocyurus chrysurus* (Usiminas, MNUFRJ-ZA-519)

GERREIDAE

*Diapterus rhombeus* (Caieira II, MNUFRJ-ZA-306);; *Gerres cinereus* (Algodão, MNUFRJ-ZA-223); Gerreidae (Algodão, MNUFRJ-ZA-485)

HAEMULIDAE

*Anisostremus virginicus* (Algodão, MNUFRJ-ZA-192); *Anisostremus* sp. (Algodão, MNUFRJ-ZA-859, MNUFRJ-ZA-692); *Haemulon aurolineatus* (Usiminas, MNUFRJ-ZA-517); *Haemulon scirius* (Algodão, MNUFRJ-ZA-184); *Haemulon steindachneri* (Algodão, MNUFRJ-ZA-220)

SPARIDAE

*Archosargus rhomboidalis* (Algodão, MNUFRJ-ZA-194); *Archosargus* sp. (Algodão, MNUFRJ-ZA-193, Ilha do Cabo Frio, MNUFRJ-ZA-873); *Diplodus* sp. (Acaiá, MNUFRJ-ZA-682, Algodão, MNUFRJ-ZA-215, MNUFRJ-ZA-217).

SCIAENIDAE

*Bairdiella ronchus* (Algodão, MNUFRJ-ZA-203); *Cynoscion* *acoupa* (Manitiba, MNUFRJ-ZA-559); *Cynoscion jamaicensis* (Acaiá, MNUFRJ-ZA-876); *Cynoscion similis* (Algodão, MNUFRJ-ZA-213); *Larimus breviceps* (Algodão, MNUFRJ-ZA-226); *Micropogonias furnieri* (Algodão, MNUFRJ-ZA-232); *Pareques acuminatus* (Manitiba, MNUFRJ-ZA-875); *Pogonias cromis* (Ponte do Girau, MNUFRJ-ZA-562, Manitiba, MNUFRJ-ZA-552); *Umbrina coroides* (Ilha do Cabo Frio, MNUFRJ-ZA-874)

MUGILIDAE

*Mugil liza* (Algodão, MNUFRJ-ZA-233); *Mugil* sp. (Manitiba, MNUFRJ-ZA-550)

LABRIDAE

*Bodianus rufus* (Acaiá, MNUFRJ-ZA-828, Ilha do Cabo Frio, MNUFRJ-ZA-872)

SCARIDAE

*Scarus* sp. (Algodão, MNUFRJ-ZA-993, MNUFRJ-ZA-496, Acaiá, MNUFRJ-ZA-724, MNUFRJ-ZA-674); *Sparisoma* sp. (Usiminas, MNUFRJ-ZA-436, MNUFRJ-ZA-428, Acaiá, MNUFRJ-ZA-720, MNUFRJ-ZA-730, MNUFRJ-ZA-666,)

TRICHIURIDAE

*Trichiurus lepturus* (Acaiá, MNUFRJ-ZA-746)

SCOMBRIDAE

*Katsowonus pelanis* (Acaiá, MNUFRJ-ZA-705, Acaiá, MNUFRJ-ZA-710, MNUFRJ-ZA-701); *Scomberomorus* sp. (Acaiá, MNUFRJ-ZA-701, MNUFRJ-ZA-732, MNUFRJ-ZA-742, MNUFRJ-ZA-786, MNUFRJ-ZA-793)

SPHYRAENIDAE

*Sphyraena guachancho* (Algodão, MNUFRJ-ZA-236); *Sphyraena barracuda* (Usiminas*,* MNUFRJ-ZA-395)

ISTIOPHORIDAE

*Ictiophorus albicans* (Usiminas, MNUFRJ-ZA-471)

EPHIPPIDAE

*Chaetodipterus faber* (Saquarema, MNUFRJ-ZA-509, MNUFRJ-ZA-656)

HYPORHAMPHIDAE

*Hyporhamphus unifasciatus* (Algodão, MNUFRJ-ZA-2230)

TETRAODONTIDAE

*Lagocephalus laevigatus* (Acaiá, MNUFRJ-ZA-679)

DIODONTIDAE

*Chilomycterus spinosus* (Algodão, MNUFRJ-ZA-487)

*Diodon* sp. (Usiminas, MNUFRJ-ZA-429)
