# Supplementary material for: The Path towards Endangered Species: Prehistoric Fisheries in Southeastern Brazil
Source: PLoS One. 2016 Jun 29;11(6):e0154476. doi: 10.1371/journal.pone.0154476 (PMC4939631; doi:10.1371/journal.pone.0154476)
Supplement: S3 Appendix — A. Ichthyological collection, Departamento de Biologia Animal e Vegetal—Instituto de Biologia, Universidade do Estado do Rio de Janeiro (UERJ)—curators: Ulisses Leite Gomes, Maisa da Cruz Lima, Cristina Paragó, Alexandra Pinto Quintans. B. Ichthyological collection, Otoliths, Departamento de Biologia Marinha—Instituto de Biologia, Universidade Federal Fluminense (UFF)–curator: Orangel Aguilera. C. Ichthyological collection, Dry skeletons, Departamento de Biologia Marinha—Instituto de Biologia, Universidade Federal Fluminense (UFF)–curators: Orangel Aguilera. (DOC) [file pone.0154476.s003.doc]

**S3 Appendix. Modern material.**

A. Ichthyological collection, Departamento de Biologia Animal e Vegetal – Instituto de Biologia, Universidade do Estado do Rio de Janeiro (UERJ) – curators: Ulisses Leite Gomes, Maisa da Cruz Lima, Cristina Paragó, Alexandra Pinto Quintans.

**Reference:** Gomes UL, Lima MC, Paragó C, Quintans AP. Catálogo das Coleções Ictiológicas do Departamento de Biologia Animal e Vegetal. 1st ed. Gráfica UERJ, Rio de Janeiro, Brasil, 1997.

CHONDRICHTHYES

ODONTASPIDIDAE

*Odontaspis noronhai* (AC.DBAV.UERJ.721), *Carcharias taurus* (AC.DBAV.UERJ.069)

ALOPIDAE

*Alopias superciliosus* (AC.DBAV.UERJ.137)

LAMNIDAE

*Carcharodon carcharias* (AC.DBAV.UERJ.290), *Isurus oxyrinchus* (AC.DBAV.UERJ.139), *Lamna nasus* (C.DBAV.UERJ.1118), *Isurus paucus* (AC.DBAV.UERJ.891)

CARCHARHINIDAE

*Carcharhinus acronotus* (C.DBAV.UERJ.1542), *Carcharhinus altimus* (AC.DBAV.UERJ.691), *Carcharhinus brachyurus* (AC.DBAV.UERJ.134), *Carcharhinus brevipinna* (AC.DBAV.UERJ.085), *Carcharhinus limbatus* (C.DBAV.UERJ.666), *Carcharhinus obscurus* (C.DBAV.UERJ.1123), *Carcharhinus plumbeus* (AC.DBAV.UERJ.083), *Carcharhinus falciformes* (AC.DBAV.UERJ.057), *Carcharhinus leucas* (AC.DBAV.UERJ.833), *Carcharhinus maou* (C.DBAV.UERJ.447), *Carcharhinus signatus* (AC.DBAV.UERJ.889), *Carcharhinus porosus* (C.DBAV.UERJ.865), *Galeocerdo cuvieri* (AC.DBAV.UERJ.360), *Negaprion brevirostris* (C.DBAV.UERJ.228), *Rhizoprionodon lalandii* (C.DBAV.UERJ.664), *Rhizoprionodon porosus* (C.DBAV.UERJ.371), *Prionace glauca* (C.DBAV.UERJ.773)

SPHYRNIDAE

*Sphyrna lewini* (AC.DBAV.UERJ.099), *Sphyrna mokarran* (AC.DBAV.UERJ.933), *Sphyrna tiburo* (AC.DBAV.UERJ.528), *Sphyrna tudes* (C.DBAV.UERJ.1121), *Sphyrna zygaena* (AC.DBAV.UERJ.100)

PRISTIDAE

*Pristis pectinata* (AC.DBAV.UERJ.900)

DASYATIDAE

*Dasyatis centroura* (C.DBAV.UERJ.785), *Dasyatis guttata* (AC.DBAV.UERJ.81), *Dasyatis sayi* (AC.DBAV.UERJ.705), *Dasyatis violacea* (AC.DBAV.UERJ.885)

MYLIOBATIDAE

*Aetobatus narinari* (C.DBAV.UERJ.707), *Myliobatis freminvillei* (C.DBAV.UERJ.391), *Myliobatis goodei* (C.DBAV.UERJ.390), *Rhinoptera bonasus* (AC.DBAV.UERJ.922), *Rhinoptera brasiliensis* (AC.DBAV.UERJ.770)

B. Ichthyological collection, Otoliths, Departamento de Biologia Marinha– Instituto de Biologia, Universidade Federal Fluminense (UFF) – curator: Orangel Aguilera.

OSTEICHTHYES

ALBULIDAE

*Albula* *vulpes* (UFF-ZO-101), *Albula* *nemoptera* (UFF-ZO-102)

ARIIDAE

*Cathorops* *wryu* (UFF-ZO-107), *Bagre Bagre* (UFF-ZO-128), *Bagre* *marinus* (UFF-ZO-108), [*Sciades herzbergii*](http://www.fishbase.org/summary/951) (UFF-ZO-110), *Amphiarius* *rugispinis* (UFF-ZO-114), *Genidens* *genidens* (UFF-ZO-116), *Notarius* *grandicassis* (UFF-ZO-117), *Genidens* *planifrons* (UFF-ZO-119), *Genidens* *barbus* (UFF-ZO-120), [*Aspistor* *quadriscutis*](http://www.fishbase.org/summary/956) (UFF-ZO-123), *Sciades* *parkeri* (UFF-ZO-125)

HOLOCENTRIDAE

*Sargocentrum* sp. (UFF-ZO-130)

TRIGLIDAE

*Prionotus* *phyryas* (UFF-ZO-098), *Prionotus* *roseus* (UFF-ZO-099), *Prionotus* *punctatus* (UFF-ZO-100)

CENTROPOMIDAE

*Centropomus* *undecimalis* (UFF-ZO-046), *Centropomus* *ensiferus* (UFF-ZO-047)

SERRANIDAE

*Hypoplectrus* *puella* (UFF-ZO-027), *Diplectrum* *formosum* (UFF-ZO-028), *Epinephelus* *itajara* (UFF-ZO-029), *Epinephelus* *morio* (UFF-ZO-030), *Epinephelus* *migritus* (UFF-ZO-031), *Serranus* *phoebe* (UFF-ZO-032), *Epinephelus* *niveatus* (UFF-ZO-033), *Diplectrum* *radiale* (UFF-ZO-034), *Myteroperca* *phenax* (UFF-ZO-035), *Diplectrum* *bivittatum* (UFF-ZO-036), *Myteroperca* *acutirostris* (UFF-ZO-037), *Serranus* *atrobranchus* (UFF-ZO-038)

CORYPHAENIDAE

*Coryphaena* *hippurus* (UFF-ZO-085), *Symphurus* *diomedianus* (UFF-ZO-086)

CARANGIDAE

*Decapterus* *punctatus* (UFF-ZO-064), *Chloroscombrus* *chrysurus* (UFF-ZO-065), *Trachurus* *lathami* (UFF-ZO-066), *Selene* *vomer* (UFF-ZO-067), *Elegatis* *bipinnulata* (UFF-ZO-068), *Caranx* *latus* (UFF-ZO-069), *Selene* *setapinnis* (UFF-ZO-070), *Selene* *brownii* (UFF-ZO-071), *Selar* *crumenophthalmus* (UFF-ZO-072), *Decapterus* *macarellus* (UFF-ZO-073), *Trachinotus* *carolinus* (UFF-ZO-074), *Oligoplites* *saurus* (UFF-ZO-075), *Selar* *crumenophthalmus* (UFF-ZO-076)

LUTJANIDAE

*Rhomboplites* *aurorubens* (UFF-ZO-053), *Pristipomoides* *macrophthalmus* (UFF-ZO-054), *Lutjanus* *synagris* (UFF-ZO-055), *Etelis* *aculatus* (UFF-ZO-056), *Pristipomoides* *aquilonaris* (UFF-ZO-057), *Ocyurus* *chrysurus* (UFF-ZO-058)

GERREIDAE

*Diapterus* *olisthostomus* (UFF-ZO-039), *Diapterus* *auratus* (UFF-ZO-040), *Eucinostomus* *argenteus* (UFF-ZO-041), *Diapterus* *rhombeus* (UFF-ZO-042), *Gerres* *cinereus* (UFF-ZO-043), *Eucinostomus* *gula* (UFF-ZO-044), *Eucinostomus* *argenteus* (UFF-ZO-045)

HAEMULIDAE

*Anisostremus* *virginicus* (UFF-ZO-077), *Anisostremus* *surinamensis* (UFF-ZO-078), *Ortopristis* *ruber* (UFF-ZO-079), *Haemulon* *steindachneri* (UFF-ZO-080), *Haemulon* *scirius* (UFF-ZO-081), *Haemulon* *melanurum* (UFF-ZO-082), *Conodon* *mobilis* (UFF-ZO-083), *Haemulon* *aurolineatus* (UFF-Z0-084)

SPARIDAE

*Pagrus* *pagrus* (UFF-ZO-059), *Archosargus* *romboidalis* (UFF-ZO-060), *Calamus* *penna* (UFF-ZO-061), *Diplodus* sp. (UFF-ZO-062), *Sparus* sp. (UFF-ZO-063)

SCIAENIDAE

*Larimus breviceps* (UFF-ZO-001), *Stellifer rastrifer* (UFF-ZO-002), *Macrodon ancylodon* (UFF-ZO-003), *Steliffer chaoi* (UFF-ZO-004), *Nebris microps* (UFF-ZO-005), *Ctenosciaena* *gracilicirrhus* (UFF-ZO-006), *Bairdiella* *santaeluciae* (UFF-ZO-007), *Pachypops* *grummiens* (UFF-ZO-008), *Cynoscion* *leiarchus* (UFF-ZO-009), *Cynoscion* *acoupa* (UFF-ZO-010), *Umbrina* *coroides* (UFF-ZO-011), *Parenques* *acuminatus* (UFF-ZO-012), *Sciaena* *trewavasae* (UFF-ZO-013), *Sciaena* *bathytatos* (UFF-ZO-014), *Isopistus* *parvipinnis* (UFF-ZO-015), *Stellifer* *naso* (UFF-ZO-016), *Micropogonias* *furnieri* (UFF-ZO-018), *Menticirrus* *americanus* (UFF-ZO-019), *Cynoscion* *microlepidotus* (UFF-ZO-021), *Pogonias* *cromis* (UFF-ZO-022), *Bairdiella* *ronchus* (UFF ZO-023), *Cynoscion* *jamaiscensis* (UFF-ZO-024), *Larimus* *breviceps* (UFF-ZO-025), *Cynoscion* *similis* (UFF-ZO-026)

MUGILIDAE

*Mugil* *lisa* (UFF-ZO-090), *Mugil* *curema* (UFF-ZO-091)

LABRIDAE

*Bodianus rufus* (UFF-ZO-131)

SCARIDAE

*Nicholsina* *usta* (UFF-ZO-049), *Sparisoma* *griseorubrum* (UFF-ZO-050), *Sparisoma* *rubripinne* (UFF-ZO-051), *Scarus* sp. (UFF-ZO-052)

TRICHIURIDAE

*Trichiurus* *lepturus* (UFF-ZO-092)

SCOMBRIDAE

*Katsowonus* *pelanis* (UFF-ZO-093), *Scomberomorus* sp. (UFF-ZO-094), *Euthynnus* *aletteratus* (UFF-ZO-095)

ISTIOPHORIDAE

*Ictiophorus* *albicans* (UFF-ZO-096)

EPHIPPIDAE

*Chaetodipterus* *faber* (UFF-ZO-097)

SPHYRAENIDAE

*Sphyraena* *barracuda* (UFF-ZO-087), *Sphyraena* *picudilla* (UFF-ZO-088), *Sphyraena* *guachancho* (UFF-ZO-089)

HYPORHAMPHIDAE

*Hyporhamphus unifasciatus* (UFF-ZO-105)

DIODONTIDAE

*Diodon* *holacanthus* (UFF-ZO-103), *Chilomycterus* *spinosus* (UFF-ZO-104)

C. Ichthyological collection, Dry skeletons, Departamento de Biologia Marinha – Instituto de Biologia, Universidade Federal Fluminense (UFF) – curators: Orangel Aguilera.

OSTEICHTHYES

ARIIDAE

*Genidens genidens* (UFF-ZO-155) (1 ind.), *Aspistor quadriscutis* (UFF-ZO-159) (1 ind.)

CENTROPOMIDAE

*Centropomus parallelus* (UFF-ZO-139) (1 ind.)

CORYPHAENIDAE

*Coryphaena hippurus* (UFF-ZO-146) (1 ind.)

CARANGIDAE

*Caranx crysos* (UFF-ZO-143) (1 ind.), *Caranx latus* (UFF-ZO-144) (1 ind.), *Parona signata* (UFF-ZO-152) (1 ind.)

LUTJANIDAE

*Lutjanus synagris* (UFF-ZO-153) (1 ind.)

GERREIDAE

*Diapterus rhombeus* (UFF-ZO-138) (1 ind.)

HAEMULIDAE

*Orthopristis ruber* (UFF-ZO-137) (1 ind.), *Haemulon steidachneri* (UFF-ZO-149) (1 ind.)

EPHIPPIDAE

*Chaetodipterus faber* (UFF-ZO-134) (1 ind.)

SPHYRAENIDAE

*Sphyraena guachando* (UFF-ZO-145) (1 ind.)

SCIAENIDAE

*Menticirrus americanus* (UFF-ZO-141) (1 ind.), *Micropogonias furnieri* (UFF-ZO-148) (1 ind.)

MUGILIDAE

*Mugil curema* (UFF-ZO-147) (1 ind.)

TETRADONTIDAE

*Lagocephalus laevigatus* (UFF-ZO-133) (1 ind.)

DIODONTIDAE

*Chylomycterus spinosus* (UFF-ZO-132) (1 ind.)

ACANTHURIDAE

*Acanthurus chirurgus* (UFF-ZO-157) (1 ind.)

DACTYLOPTERIDAE

*Dactylopterus volitans* (UFF-ZO-136) (1 ind.)

HOLOCENTRIDAE

*Holocentrus adscensionis* (UFF-ZO-151) (1 ind.)

KYPHOSIDAE

*Kyphosus* *septatrix* (UFF-ZO-135) (1 ind.)

MONACANTHIDAE

*Cantherhines pullus* (UFF-ZO-140) (1 ind.), *Monacanthus hispidus* (UFF-ZO-150) (1 ind.)

MALACANTHIDAE

*Lopholatilus villarii* (UFF-ZO-142) (1 ind.)

PRIACANTHIDAE

*Heteropriacanthus cruentatus* (UFF-ZO-156) (1 ind.)

PINGUIPEDIDAE

*Pseudopercis numida* (UFF-ZO-158) (1 ind.)

PIMELODIDAE

*Abudefduf saxatilis* (UFF-ZO-154) (1 ind.)
