# Supplementary material for: The Path towards Endangered Species: Prehistoric Fisheries in Southeastern Brazil
Source: PLoS One. 2016 Jun 29;11(6):e0154476. doi: 10.1371/journal.pone.0154476 (PMC4939631; doi:10.1371/journal.pone.0154476)
Supplement: S4 Appendix — Artwork by Eduardo Agelvis. (DOCX) [file pone.0154476.s004.docx]

**S4 Appendix. The unknown prehistoric fishing.** Artwork by Eduardo Agelvis.

**
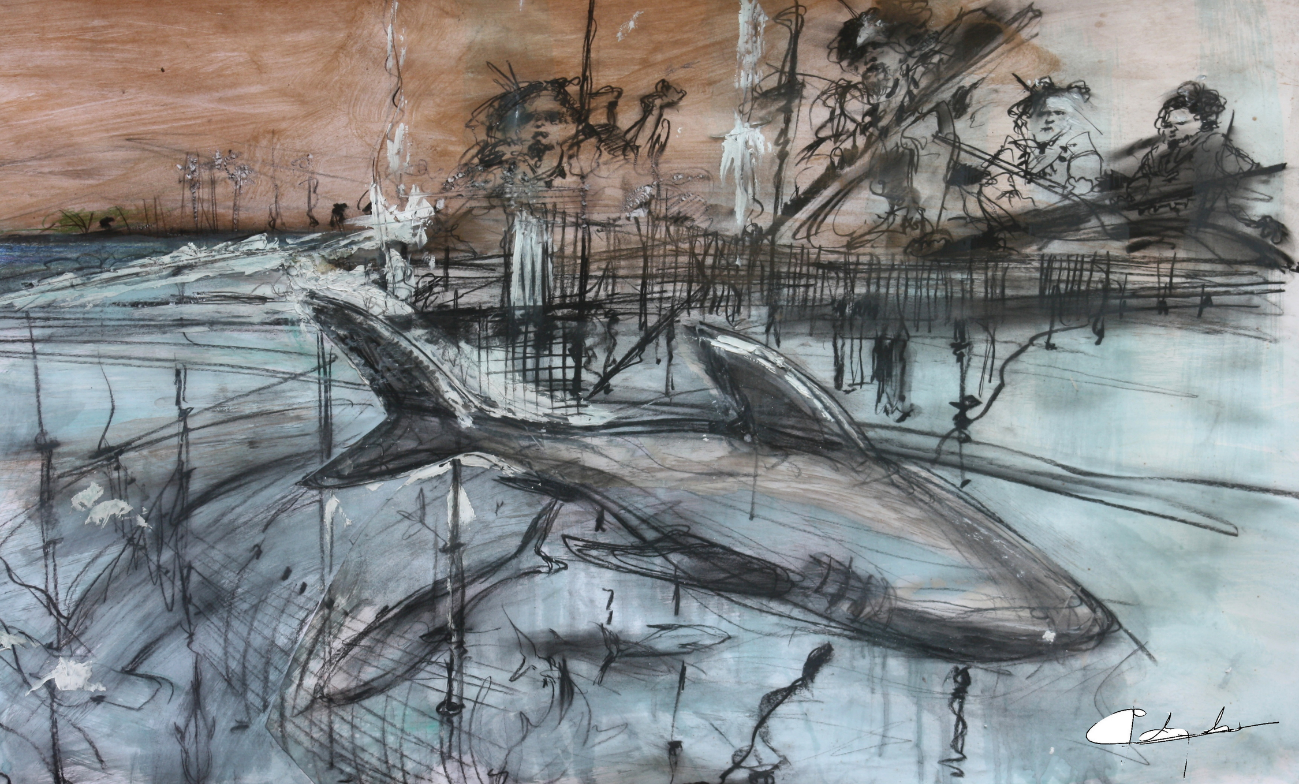
**

**Fig 1. Restoration of prehistoric fishing at the late Holocene times.** Artwork by Eduardo Agelvis.
